# Changes in the Responsiveness of the Hypothalamic-Pituitary-Gonadal Axis to Kisspeptin-10 Administration during Pubertal Transition in Boys

**DOI:** 10.1155/2018/1475967

**Published:** 2018-06-26

**Authors:** Ghulam Nabi, Hamid Ullah, Suliman Khan, Fazal Wahab, Pengfei Duan, Rahim Ullah, Lunguang Yao, Muhammad Shahab

**Affiliations:** ^1^Laboratory of Reproductive Neuroendocrinology, Department of Animal Sciences, Faculty of Biological Sciences, Quaid-i-Azam University, Islamabad 45320, Pakistan; ^2^Institute of Hydrobiology, Chinese Academy of Sciences, Wuhan 430072, China; ^3^German Primate Center, Göttingen, Germany; ^4^China-UK-NYNU-Research Joint Laboratory of Insects Biology, Nanyang Normal University, Nanyang, Henan, China; ^5^Department of Endocrinology, Children's Hospital, Zhejiang University School of Medicine, Hangzhou, China

## Abstract

In human, no studies are available regarding changes in kisspeptin1 receptor (KISS1R) sensitivity during pubertal transition. In this study, healthy boys were classified into 5 Tanner stages of puberty (*n* = 5/stage). Human kisspeptin-10 was administered to boys at each Tanner stage and to adult men (*n* = 5) as an IV bolus for comparison. Serial blood samples were collected for 30 min pre- and 120 min post-kisspeptin injection periods at 30 min interval for measuring plasma LH and testosterone levels. There was insignificant effect of kisspeptin on LH and testosterone levels in boys of Tanner stages I–III. At Tanner stage IV, the effect of kisspeptin on plasma LH was insignificant. However, a paired *t*-test on a log-transformed data showed a significant (*P* < 0.05) increase in mean peak post-kisspeptin testosterone level. In Tanner stage V, a significant (*P* < 0.05) increase was observed in mean post-kisspeptin peak LH level as compared to the mean basal LH value. Post-kisspeptin plasma testosterone levels were also significantly (*P* < 0.05) increased as compared to the pre-kisspeptin level in Tanner stage V. Our data suggest that sensitivity of KISS1R on GnRH neurons with reference to LH stimulation in boys develops during the later part of puberty reaching to adult level at Tanner stage V. This trial is registered with WHO International Clinical Trial Registration ID NCT03286517.

## 1. Introduction

Pubertal initiation, in human biology, is one of the greatest mysteries as very little information is available on physiology, maturation, and secretion of gonadotropin-releasing hormone (GnRH) as well as key elements involved in pubertal progression. In recent years, the role of kisspeptin and G-protein coupled receptor 54 (GPR54 aka KISS1R) has been increasingly indicated for the control of GnRH secretion and pubertal transition. In human patients, a mutation of *KISS1R* at different sites results in either precocious [1] delayed or absent puberty [2–8]. Similarly, pubertal failure and a low level of sex steroids, gonadotropins, and immature reproductive organs were noticed in mice with a genetically targeted deletion in either kisspeptin receptor *(Kiss1r)* or kisspeptin 1 *(Kiss1)* [3, 9, 10]. Both KISS1 and KISS1R are crucial gatekeepers of GnRH neuron, triggering neuroendocrine puberty after the resurgence of GnRH pulsatility [11, 12]. This finding is supported by the significantly higher levels of kisspeptin reported in girls with central precocious puberty (CCP) [13, 14]. Both pharmacological and physiological studies have confirmed that kisspeptin is the most potent GnRH secretagogue [15]. Endogenous as well as exogenous kisspeptin stimulated GnRH enter into the hypophyseal portal circulation in a pulsatile manner, acting on the pituitary gonadotrophs to regulate steroidogenesis, gametogenesis, and thus pubertal onset and adult fertility by secreting gonadotropins [16–19]. During the human fetal and neonatal stage, the GnRH pulsatility starts; this is suppressed in early childhood (juvenile pause) until adolescence, when the resurgence of the GnRH pulsatility is established, stimulating reproductive maturation and pubertal development [20–22]. During a juvenile pause, suppression of GnRH pulsatility until initiation of puberty is considered as a hypothetical neurobiological brake, which may be accounted for by either the imposition of the loss of an inhibitory input or stimulatory input to GnRH neurons [23]. According to Terasawa and Fernandez, juvenile pause is due to the inhibitory action of gamma-amino butyric acid (GABA) [20], but according to El-Majdoubi and his colleagues, neuropeptide Y (NPY) is responsible for this central inhibition [24]. In a nutshell, the exact pubertal trigger in primates remains a mystery as no information is available on control of GABA [25] and NPY or whether additional or alternative neuronal substrates or somatic cues [25–28] are involved in the upstream control of the GnRH pulse generation.

In boys, physiological pubertal maturation occurs in 5 stages called Tanner stages (I–V) [29]. Recently, leptin receptors were also reported in the arcuate (ARC) kisspeptin neuron, indicating that leptin regulation of gene expression is likely to occur directly on kisspeptin neurons, facilitating the induction of puberty [30, 31]. Kisspeptin is a fundamental regulator of GnRH both in puberty and adulthood [32]. The hypothalamic expression of *kiss1* in rats and monkeys increased during the progression of puberty [33, 34], while a high *KISS1R* mRNA level was observed in female monkeys during the pubertal progression [34]. *Kiss1r* expression in both sexes of rats and female mice was found to be higher in adulthood as compared to juvenile period [11, 33]. Kisspeptin release during puberty in human increases because a serum kisspeptin level in Korean girls with central precocious puberty was found significantly higher as compared to age-matched prepubertal control group [35]. No studies in humans are available regarding KISS1R signaling that whether expression of *KISS1* or KISS1R sensitivity increases during the pubertal transition. The specific objective of this study, therefore, was to investigate the sensitivity of KISS1R by determining the responsiveness of GnRH-induced LH and testosterone secretion from the pituitary gonadotrophs and testes to kisspeptin administration across the pubertal stages and adult group through measuring plasma luteinizing hormone (LH) and testosterone concentrations. This study would help us to determine the role of kisspeptin signaling in the activation of HPG axis during puberty onset in boys through GnRH and pituitary gonadotroph responsiveness.

## 2. Materials and Methods

### 2.1. Ethical Approval

Ethical approval for the study was obtained from the Research Ethics Committee of Quaid-i-Azam University (letter number DFBS-2014/3204) and also from the office of the Medical Superintendent, District Head Quarter Hospital, Batkhela, Khyber Pakhtunkhwa (letter number 2588/G-I). This clinical trial is performed in accordance with the WHO guidelines and regulations and is also registered in the WHO International Clinical Trial Registry Platform under the ID NCT03286517.

### 2.2. Study Participants

Twenty-five healthy male children and five adult men were recruited as per inclusion and exclusion criteria. The children were classified into five pubertal stages called Tanner stages, according to the Feingold [36] criteria (inclusion). Each Tanner stage comprised of five participants. Anthropometric data of the adult group, as well as both genital and anthropometric data of all the Tanner groups, are shown in Table 1. All these participants were a resident of Lower Dir District, a district of Khyber Pakhtunkhwa Province, Pakistan. The participants belonged to a middle class, socioeconomic group. Informed written consent was obtained from all the volunteers who participated in this study as per World Health Organization's (WHO) pro forma. Individuals with chronic illness or disorder, that is, hepatic and renal complications, epilepsy, pneumonia, asthma, orchitis, hernia, cryptorchidism, and intellectual disability, were excluded from this study.

### 2.3. Experimental Design

Each day, a given cohort of the Tanner stage was sampled before and after kisspeptin-10 administration starting from the Tanner stage I. The adult group was sampled one month later on a single day. The blood sampling was done at the Health Care Clinical Laboratory, Batkhela. All the subjects had breakfast at 0700 hr. The blood sampling was started at 09:00 a.m. and ended at 1:00 p.m. daily during a period from June 26 to August 8, 2014. Sequential blood samples (1 ml for children and 2 ml for adults) were obtained for 30 minutes pre- and 120 minutes post-kisspeptin injection periods at 30 min intervals (−30, 0, 30, 60, 90, 120). Kisspeptin-10 (metastin 45-54, Calbiochem, Darmstadt, Germany) was administered as an intravenous bolus, immediately after collecting 0 min sample. For the ease of blood sampling and kisspeptin injection, the volunteers were fitted with an infusion cannula (Farcocath; G/Ø/L: 22, 0.9, 25 mm; Farcomake for Advanced Medical Industries SAE Alexandria, Egypt) in the cephalic vein. Blood samples were collected in heparinized syringes (BD 3 ml, Luer-Lok Tip with PrecisionGlide Needle, 23G × 1TW (0.6 × 25 mm), Becton Dickinson Pakistan Pvt Ltd.). To prevent blood clotting in the cannula, heparin (heparin; 5000 IU/ml; B. Braun Melsungen AG, Melsungen, Germany) was used at the rate of 10 IU/ml in saline (0.9% NaCl) as a flushing agent. For children, kisspeptin doses were calculated according to the allometric scaling equation as [37]

(1) $$\begin{array}{c} Pchild=Padults\left(\frac{WT}{70}\right)X. \end{array}$$

In this equation, *P* represents the parameter of interest (kisspeptin-10), WT represents the individual child total body weight in kg, 70 refers to a standard human weight, and *X* is the allometric exponent. According to this equation, the dose was 9.5 *μ*g/body weight (BW), 11.50 *μ*g/BW, 12.67 *μ*g/BW, 15.11 *μ*g/BW, and 20.5 *μ*g/BW for Tanner groups I–V, respectively. For an adult, 1 *μ*g/kg kisspeptin dose was used on the basis of a previous study [38]. These doses were prepared in a hygienic environment. After preparation, these doses were immediately frozen and shortly before transportation were kept in liquid nitrogen. Before kisspeptin administration, doses were thawed, and 1 ml of sterile saline was mixed with the dose to give a dose volume of 1 ml. Blood samples were then collected and transferred to culture tubes and stored in the refrigerator at 4°C until centrifuged. The process of centrifugation was done just after the completion of blood sampling (within 2 hours) at 3000 rpm for 15 minutes (centrifuge model 800, China). Blood plasma was isolated in Eppendorf vials of 1.5 ml capacity and stored at −20°C until hormonal analysis.

### 2.4. Hormonal Analyses

An EIA for human LH (Biocheck, Foster City, CA, USA) and testosterone (AMGENIX, San Jose, USA) was used for the determination of LH and testosterone concentration in plasma according to the manufacturer's protocol and procedures. The intra-assay coefficient of variation was <9% for LH and 8.5% for testosterone. The minimal detectable concentration of human LH and testosterone by this assay was estimated to be 1 mIU/ml and 0.05 ng/ml, respectively.

### 2.5. Statistical Analyses

Changes in mean plasma LH and testosterone concentrations were assessed by one-way ANOVA followed by a post hoc (Tukey) test in each group. A comparison of mean pre-LH and testosterone versus mean post-LH and testosterone was made by paired *t*-test. Comparison of plasma LH and testosterone across the groups was determined by using one-way ANOVA followed by a post hoc (Tukey) test. All data are presented as mean ± SEM. A *P* < 0.05 indicated the significant difference. Analysis of data was done using the GraphPad Prism, version 5.01 (GraphPad Software Inc., San Diego, CA, USA).

## 3. Results

### 3.1. Effect of a Single IV Bolus Administration of Kisspeptin-10 on Plasma LH and Testosterone Levels in Boys of Different Tanner Stages and Adult Men

#### 3.1.1. Tanner Stage I

**Figure 1:**
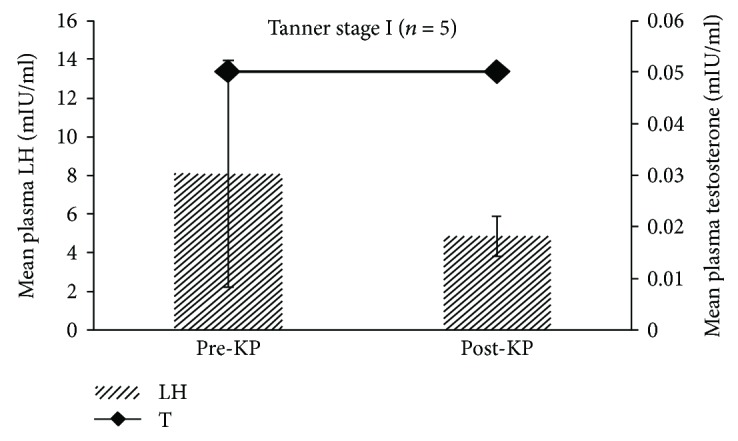
Comparison of overall mean ± SEM plasma LH and testosterone concentrations observed before and after kisspeptin administration in Tanner stage I boys. Paired *t*-test showed that post-kisspeptin plasma LH level was comparable to the pre-kisspeptin level. Testosterone level was below the range of detection in both pre- and post-kisspeptin plasma.

One-way ANOVA with repeated measures showed that there was an insignificant variation observed in mean plasma LH concentrations before and after kisspeptin-10 administrations (S1–S3). A paired *t*-test showed that the post-kisspeptin mean plasma LH levels were comparable to the pre-kisspeptin level (Figure 1). Similarly, a paired *t*-test showed an insignificant elevation in mean% LH response to kisspeptin administration over basal values. Testosterone concentration in all the boys of Tanner stage I was below the range of detection (Figure 1). Therefore, their concentration was considered 0.05 ng/ml (minimum sensitivity of the assay) (S4 and S5).

#### 3.1.2. Tanner Stage II

Like Tanner stage I boys, no significant increase in post-kisspeptin LH concentrations was reported by different statistical analysis (S6 and S7). Similarly, testosterone concentration like boys of Tanner stage I was below the range of detection. Therefore, their concentration was considered 0.05 ng/ml (minimum sensitivity of the assay) (S4 and S5).

#### 3.1.3. Tanner Stage III

Like Tanner stage I and II boys, no significant increase in post-kisspeptin LH was observed. Testosterone concentration in 2 boys of Tanner stage III was below the range of detection and like Tanner stage I and II boys were considered 0.05 ng/ml (minimum sensitivity of the assay). In the 3 remaining boys, no significant variations by one one-way ANOVA with repeated measures and no significant difference in mean pre- and post-kisspeptin as well as a mean% testosterone response to kisspeptin administration over the basal values were recorded by paired *t*-test (S8–S11).

#### 3.1.4. Tanner Stage IV

**Figure 2:**
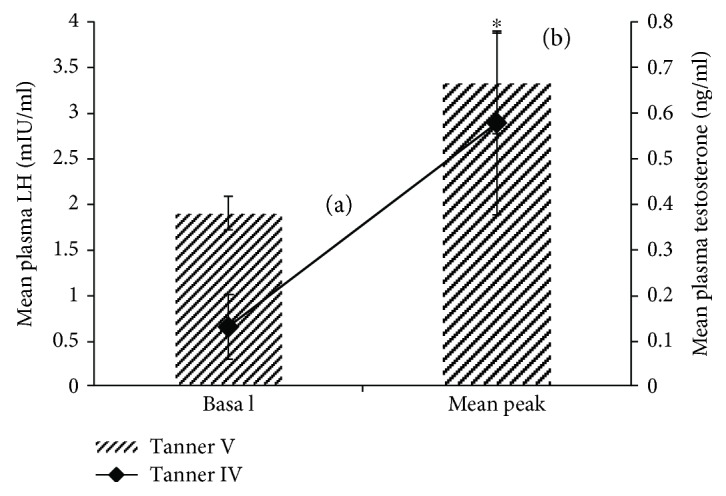
(a) Comparison of overall mean ± SEM peak testosterone concentration observed following kisspeptin-10 administration with mean overall basal values in Tanner stage IV boys. Paired *t*-test on a log-transformed data showed a significant (^∗^ *P* < 0.05) increase in mean peak testosterone level as compared to the mean basal testosterone value. (b) Comparison of overall mean ± SEM peak LH concentration observed following kisspeptin-10 administration with mean overall basal values in Tanner stage V boys. Paired *t*-test on a log-transformed data showed a significant (^∗^ *P* < 0.05) increase in mean peak LH level as compared to the mean basal LH value.

Like Tanner stages I–III, no significant increase in post-kisspeptin LH and testosterone was observed. However, in Tanner stage IV boys, a significant (*P* < 0.05) increase in the mean peak testosterone level as compared to the mean basal testosterone values was observed by paired *t*-test (Figure 2(a), S12–S15).

#### 3.1.5. Tanner Stage V

**Figure 3:**
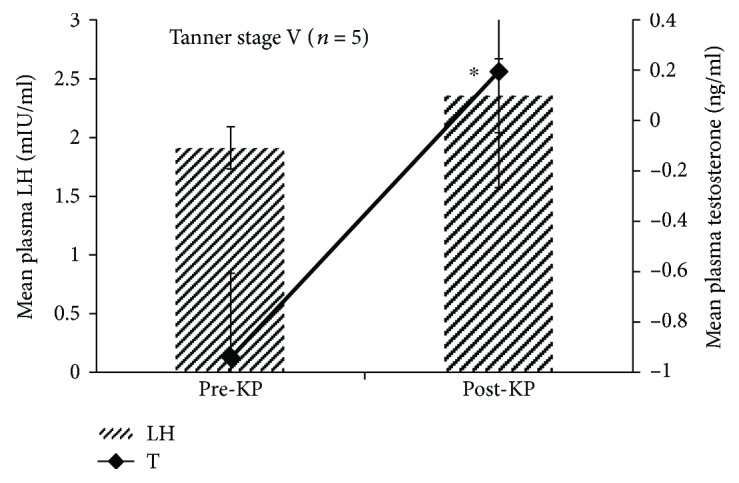
Comparison of overall mean ± SEM plasma LH and testosterone concentrations observed before and after kisspeptin administration in Tanner stage V boys. Paired *t*-test showed that the post-kisspeptin plasma LH level was insignificant to the pre-kisspeptin level. However, paired *t*-test on a log-transformed data showed that the post-kisspeptin plasma testosterone level was significantly (^∗^ *P* < 0.05) increased as compared to pre-kisspeptin level.

Like all other Tanner stages (I–IV), no significant differences in post-kisspeptin LH were observed by different statistical tests (Figure 3). However, a paired *t*-test on a log-transformed data showed a significant (*P* < 0.05) increase in the mean peak LH level as compared to the mean basal LH value (Figure 2(b)). Only in one individual, the response of plasma testosterone to kisspeptin administration was inconspicuous. One-way ANOVA with repeated measures showed no significant variations in mean plasma testosterone concentrations before and after kisspeptin-10 administrations. But a paired *t*-test on a log-transformed data showed a significant (*P* < 0.05) increase in post-kisspeptin mean plasma testosterone level (Figure 3). Similarly, mean% testosterone response to kisspeptin administration over the basal values was significant (*P* < 0.05) (S16–S19).

#### 3.1.6. Adulthood

**Figure 4:**
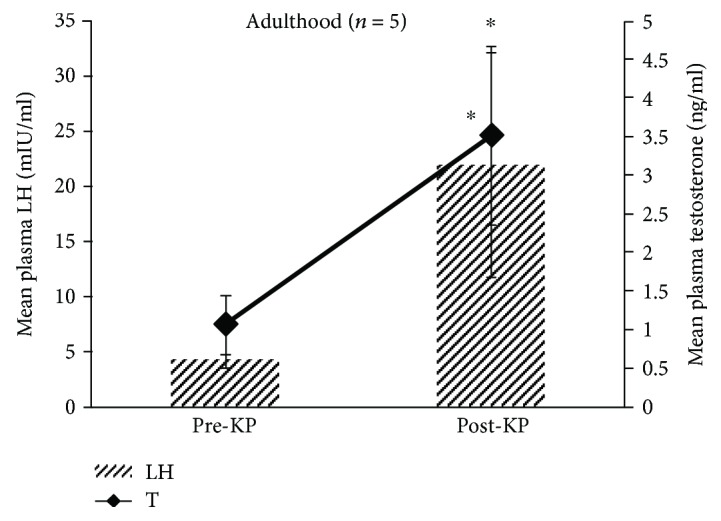
Comparison of overall mean ± SEM plasma LH and testosterone concentrations observed before and after kisspeptin-10 administration in adult men. Paired *t*-test on a log-transformed data showed significant (^∗^ *P* < 0.05) increase in both post-kisspeptin LH and testosterone levels versus pre-kisspeptin levels.

All adult men showed positive LH response to kisspeptin-10 administration with a peak plasma LH occurred in a period ranging from 30 to 90 min. One-way ANOVA with repeated measures showed significant (*P* < 0.05) variations in mean plasma LH concentrations before and after kisspeptin-10 administrations (S20 and S21). Similarly, pre- and post-kisspeptin mean plasma LH (Figure 4) and mean% LH response to kisspeptin administration over the basal values were significant (*P* < 0.05) in the adult. All adult men showed positive plasma testosterone response to kisspeptin injection. Peak plasma testosterone occurred in a period ranging from 30 to 90 min. One-way ANOVA with repeated measures showed significant (*P* < 0.05) variation in mean plasma testosterone concentrations before and after kisspeptin-10 administrations in the adult (S22 and S23). A paired *t*-test showed that the post-kisspeptin mean plasma testosterone level was significantly (*P* < 0.05) increased in adult men (Figure 4). Similarly, mean% testosterone response to kisspeptin administration over the basal values was significant (*P* < 0.05) in adult men.

There was no significant difference between Tanner stage V and adult group mean post-kisspeptin plasma LH and testosterone levels. Also, no significant difference was reported in any individual following kisspeptin administration.

#### 3.1.7. Comparisons of Kisspeptin-10 Affected LH and Testosterone Secretions across Different Tanner Stages and Adulthood

Mean post-kisspeptin LH concentration showed an increasing trend (except at Tanner stage IV) but did not vary significantly across the Tanner stages and adulthood. One-way ANOVA with repeated measures showed an insignificant elevation on mean plasma LH concentration after administration of kisspeptin-10.

**Figure 5:**
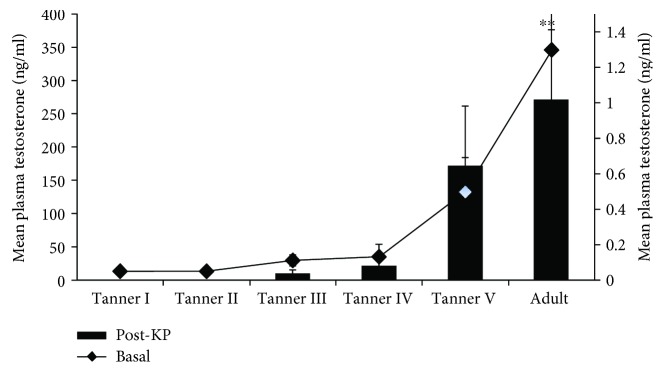
Comparison of the basal and post-kisspeptin mean ± SEM plasma testosterone level observed at different Tanner stages and adulthood (*n* = 5/stage). One-way ANOVA followed by a post hoc analysis indicated significant (^∗∗^ *P* < 0.05) elevation in the mean post-kisspeptin testosterone level at adulthood as compared to Tanner stages I–IV. No significant (*P* < 0.05) variations in the basal levels of testosterone were observed across the groups.

There was an increasing trend of mean post-kisspeptin testosterone concentration across the groups (Figure 5). One-way ANOVA with repeated measures showed a significant (*P* < 0.05) effect observed in mean plasma testosterone concentration after administration of kisspeptin-10. Further post hoc analysis indicated that a mean testosterone level observed at adulthood was significantly (*P* < 0.05) increased as compared to the Tanner stages I–IV. However, no significant (*P* < 0.05) difference was observed between Tanner stage V boys and adult men (Figure 5).

## 4. Discussion

This study describes the effect of IV bolus administration of kisspeptin-10 on circulating LH and testosterone levels during Tanner stages I–V of boys and in adult men to assess the developmental changes in the responsiveness of the HPG axis to kisspeptin in boys. Administration of kisspeptin-10 significantly increased plasma LH and testosterone level at Tanner stage V. At Tanner stage IV, the effect of kisspeptin-10 on plasma LH was insignificant, but plasma testosterone was increased significantly following a kisspeptin-10 injection. There was no significant change in plasma LH and testosterone concentrations in other Tanner stages (I–III) after kisspeptin administration. As expected in the adult group, both LH and testosterone levels were significantly increased following a kisspeptin injection. A comparison of mean post-kisspeptin plasma LH and testosterone in Tanner stage V boys and adult men showed that there was no significant difference in post-kisspeptin plasma LH and testosterone levels. Moreover, no significant change in blood pressure was observed in all study participants before and after kisspeptin administration.

In the present study, the observation of the development of significant kisspeptin stimulation of plasma LH levels later during puberty is parallel to a previously presented finding in mice where acute administration of kisspeptin in adult mice significantly stimulated LH secretion at all three doses tested (10 fmol, 0.1 pmol, and 0.1 nmol). In contrast, in juvenile mice, only the highest dose of kisspeptin (0.1 nmol) elicited an LH response [11]. Further, Han et al. [11] found that injecting 100 nmol kisspeptin-10 to juvenile, prepubertal, and adult mice progressively increased the percentage of kisspeptin-responsive GnRH neurons during pubertal development. Foregoing findings demonstrate that, over the course of postnatal development in mice, the GnRH neuronal population gradually acquires kisspeptin sensitivity. Similarly, Semaan and Kauffman [39] recently reported that LH level in female mice progressively increases during the pubertal transition because the kiss1 neuron cell number in the anteroventral periventricular nucleus (AVPV) increases steadily and substantially throughout the puberty stages reaching a peak around the time of a mean vaginal opening, whereas, in the ARC, the number of kiss1 neurons is significantly higher in older pubertal ages as compared to earlier pubertal ages. In line with the rodent data, our study also demonstrated that the responsiveness of GnRH neuron to kisspeptin administration is insignificant in the earlier stages of puberty and then reaching to an adult level at Tanner stage V. Reasons for a lack of responsiveness to kisspeptin during first 3 Tanner stages are not clear. Several factors can be postulated. This can be due to an increased inhibitory tone of central GABA, which checks the GnRH pulsatility as observed in juvenile monkeys [40]. This evidence is also supported by the observation that a long-term infusion of GABA_A_ receptor antagonist, bicuculline into the stalk-median eminence (S-ME) of juvenile female primates induces precocious puberty and first ovulation [41]. The insignificant responsiveness of GnRH neurons in these first three Tanner stages might also be due to the increased activity of hypothalamic NPY. Evidence of male rhesus monkeys suggests that mRNA and peptide level of NPY in the mediobasal hypothalamus (MBH) increases, while GnRH decreases during the juvenile period and vice versa in pubertal monkeys [24]. A lack of kisspeptin responsiveness in earlier Tanner stages can also be due to the insufficient number of KISS1R or decreased responsiveness of the KISS1R to kisspeptin. A lack of response may also be related to a dose issue; as in our study, the kisspeptin doses used were scaled, and like the study of Han et al. [11], low doses in juvenile mice were unable to stimulate GnRH neuron and that high dose in juvenile mice did elicit an LH response. Similarly, insufficient numbers of KISS1R in earlier Tanner stages contributing to a lack of kisspeptin responsiveness might be predicted in light of observations made by Shahab et al. [34] in juvenile female monkeys, where kisspeptin receptor expression was shown to be reduced. In the same experiment, Shahab et al. [34] observed a robust discharge of LH in agonadal juvenile male monkeys after kisspeptin-10 administration. However, unlike our experiment, the pituitary gonadotrophs of agonadal juvenile male monkeys were preprimed with GnRH. Furthermore, the lack of responsiveness to kisspeptin in Tanner stages I–III might also be due to the unresponsiveness of the pituitary gonadotrophs. The study of Nakada et al. [42] indicates that not only the amount of release, but also the peak concentration as well as the capacity of LH release to GnRH in the pituitary gland develop with age. It also indicates that the timing of puberty onset is also decided by the development of the capacity to secrete LH secretion in response to GnRH in the pituitary gland [42]. Therefore, further studies are needed to investigate the timing of pituitary gonadotrophs responsiveness to GnRH during pubertal transition.

In our study, at Tanner stage IV, kisspeptin-dependent LH stimulation was insignificant, but plasma testosterone level interestingly increased significantly. This increase in plasma testosterone might be due to the direct action of kisspeptin on the testes, as the *KISS1R* expression has been demonstrated in the human testes [43] which might acquire sensitivity at this stage. In rhesus monkey, kisspeptin in high doses markedly suppresses LH but amplifies testosterone production suggesting a direct action of kisspeptin on Leydig cells [44]. Similarly, according to Anjum et al. [45], kisspeptin expression in mouse testes increases from prepubertal to pubertal period and is responsible for increased circulating testosterone level and testicular weight. In the same way, Irfan et al. [46] reported that kisspeptin in adult primates exerted an intratesticular action in GnRH receptor antagonist treated monkeys. This intratesticular kisspeptin action accelerated an increased steroidogenic response towards LH/hCG stimulation.

The most important finding of the present study was that at Tanner stage V, post-kisspeptin plasma LH and testosterone level increased significantly. The acquisition of kisspeptin-dependent LH stimulation at Tanner stage V might be due to an increased expression of *KISS1R* at this stage. Such a notion is supported by the observation that *KISS1R* expression in the MBH of female monkey increases during the transition from juvenile to midpubertal stage [34]. Another reason of the specific acquisition of kisspeptin-dependent LH stimulation at Tanner stage V might be increased sensitivity of KISS1R as has been observed in mice from juvenile to prepubertal and adult stage [11]. Overall, our results suggest that kisspeptin stimulation of GnRH neurons developed at the time of Tanner stage V and is then maintained in adults.

The mechanisms underlying establishment of kisspeptin responsiveness of the GnRH neuron during Tanner stage V are not clear. One possibility can be that this happens due to changes in body composition. In our study, the BMI of participants increased progressively from Tanner stage IV to adult. Similarly, according to Mann et al. [47], leptin level increases with age during childhood and adolescence in both sexes. Leptin might act both at the hypothalamic level (as more than 40% of kisspeptin neurons have been demonstrated to express leptin receptors) [48, 49] and testicular level (as the testes express leptin receptors) [50]. These changes in leptin might potentially serve as a metabolic barometer that informs the central nervous system that energy reserves are adequate to support pubertal development [47, 51, 52]. In the present study, at Tanner stage V, the boys are achieving sufficient adiposity and may secrete sufficient leptin to stimulate hypothalamic kisspeptin signaling. This is because leptin has been reported to increase *Kiss1* mRNA levels in the murine hypothalamus [53], the sheep ARC, and the preoptic area (POA) [54] as well as in primary cultures of human fetal GnRH-secreting neuroblasts [55]. Further, Palmert et al. [56] observed high leptin concentration in girls with central precocious puberty (CCP) as compared to age-matched healthy children. Altogether, these data provide evidence for a tenable neuroendocrine *leptin-kisspeptin-GnRH* pathway, whereby sufficient levels of leptin would allow proper maturation and function of GnRH neurons and, hence, of the HPG axis through kisspeptin.

Although kisspeptin signaling is involved in regulating reproduction in a variety of paradigms, recently, it has been suggested that it may not play a regulatory role in controlling the timing of puberty per se [57]. This notion tends to support our data. In the higher primates, a resurgence of pulsatile GnRH release is considered obligatory for the onset of puberty [57]. This increased GnRH release is subsequently followed by an increased nighttime release of LH. In our data, significant kisspeptin responsiveness of the hypothalamic-pituitary unit developed only at Tanner stage V. Previous systematic studies of the pubertal ontogeny of LH secretion demonstrated that in normal boys, a first significant neuroendocrine LH peak occurred at Tanner stage II followed by a second peak at Tanner stage IV and a third peak at Tanner stage V, suggesting that real initiation of neuroendocrine puberty in boys occurs likely at Tanner stage II. Increases in testicular volume were also significant between Tanner stages I and II and Tanner stage III and Tanner stages IV and V [58]. Similarly, Albertson-Wikland et al. [59] found a marked increase in all the parameters of LH levels (mean level, maximum level, baseline, number of peaks, and mean peak amplitude) during the Tanner stage III. Foregoing observations suggest that GnRH-dependent LH stimulation, which is very crucial for the initiation of puberty, occurs before Tanner stage V. Our own finding though based on a very limited number of basal samples (2/stage) indicates that there was an increasing pattern of circulating LH level from Tanner stages I–III. It is therefore likely that a real neuroendocrine initiation of puberty in boys might occur at Tanner stage III, and that kisspeptin signaling does not play a role in this critical event. Kisspeptin signaling develops first at Tanner stage V and then becomes fundamentally important in the regulation of the reproductive axis in adulthood.

Significantly elevated levels of plasma LH and testosterone in response to kisspeptin in adult men observed in the present study are in line with previously reported studies that, in the adult men, infusion of kisspeptin-54 significantly stimulated LH, FSH, and testosterone secretion [32]. In the same way, an intravenous bolus administration of kisspeptin-10 in adult men resulted in a rapid and dose-dependent rise in serum LH concentration, with maximal stimulation at 1 *μ*g/kg. Overall kisspeptin-10 boluses potently evoked LH secretion in men, and continuous infusion increased testosterone, LH pulse frequency, and pulse size [38]. Similarly, in healthy women during the preovulatory phase of the menstrual cycle, intravenous bolus administration of kisspeptin-10 resulted in 4-fold increase in plasma LH and FSH [18]. An acute subcutaneous administration of kisspeptin-54 stimulated a potential increase in serum LH of over 20-fold in women with hypothalamic amenorrhea [60]. Kisspeptin acts directly on the GnRH neuron via KISS1R to trigger GnRH secretion, and that GnRH then acts directly on the pituitary to trigger gonadotropin release [32]. Similarly, it has been demonstrated that GnRH antagonists can block the kisspeptin-induced increase in gonadotropin secretion [34, 61], suggesting that kisspeptin-dependent gonadotropin secretion largely reflects direct activation of GnRH neurons and not pituitary gonadotrophs.

A comparison of the post-kisspeptin plasma LH and testosterone in the present study between Tanner stage V boys and adult men showed that the responsiveness of GnRH neuron to kisspeptin was established at Tanner stage V which then remained the same at adulthood. This finding is in agreement with the result of Carabulea et al. [62] who found that LH and testosterone level in boys progressively increases from the Tanner stages I to V reaching to adult level in Tanner V.

## 5. Conclusions

In summary, this study describes that an IV bolus administration of kisspeptin-10 significantly increases a plasma LH and testosterone secretion at only Tanner stage V. At Tanner stage IV, kisspeptin-10 has an insignificant effect on plasma LH but increases plasma testosterone significantly. In adults, kisspeptin has a significant effect on both plasma LH and testosterone. Further, in Tanner stage V and adult group, there was no significant difference in post-kisspeptin plasma LH and testosterone. The present data suggest that the sensitivity of kisspeptin receptors with reference to LH stimulation in the boys develops during the later part of puberty reaching to an adult level at Tanner stage V. Similarly, Leydig cells in the first three Tanner stages are totally insensitive to kisspeptin stimulation indirectly or directly. KISS1R sensitivity develops at Tanner stage IV reaching to a significant level at Tanner stage V which then remains the same during adulthood. Our data also suggest that pubertal onset is independent of kisspeptin signaling. Kisspeptin is not involved in triggering pubertal onset, but rather acts as an integral component of the hypothalamic GnRH pulse-generating mechanism causing a further intermittent increase in the secretion of GnRH which is essential for subsequent pubertal maturation of the HPG axis.

## Figures and Tables

**Table 1:** Anthropometric and genital data of all the Tanner groups and adult men.

Groups	Tanner I	Tanner II	Tanner III	Tanner IV	Tanner V	Adult
Age (years)	7.06 ± 0.19	9.6 ± 0.61	11.9 ± 0.55	14 ± 0.16	15.5 ± 0.22	25.80 ± 0.37
Body weight (kg)	20.2 ± 1.01	26 ± 1.18	29.60 ± 1.63	37.40 ± 1.24	56.4 ± 2.29	61.20 ± 1.15
Height (foot and inch)	3.62 ± 0.13	4.3 ± 0.10	4.4 ± 0.09	5 ± 0.15	5.46 ± 0.06	5.54 ± 0.08
Waist (inch)	22.4 ± 0.39	24 ± 0.54	25 ± 0.54	27.8 ± 0.73	31.1 ± 1.14	
BMI (kg/m^2^)	17.33 ± 0.71	15.47 ± 0.25	16.95 ± 0.72	18.93 ± 1.31	21.04 ± 1.09	22.06 ± 0.63
Penile length (cm)	3.06 ± 0.11	3.8 ± 0.20	4.06 ± 0.31	4.6 ± 0.36	7.6 ± 0.59	
Total testicular volume (ml)	2.51 ± 0.84	2.68 ± 0.72	5.04 ± 1.71	6.12 ± 1.73	23.28 ± 0.97	
Pubic hair	VH^∗^	SD^∗^	PH^∗^	HD^∗^	HD^∗^	
Scrotum	SS^∗^	ER^∗^	IG^∗^	DD^∗^	SC^∗^	

VH^∗^ = vellus hair appears over the pubes; SS^∗^ = scrotal sac has a size and proportion similar to those seen in early childhood; SD^∗^ = sparse development of long pigmented downy hairs especially at the base of the penis; ER^∗^ = enlargement and reddening of the scrotum; PH^∗^ = pubic hair is considerably darker, curlier, and coarser. These hairs spread over the junction of pubes; IG^∗^ = increased growth of the scrotum; HD^∗^ = hair distributions are of adult type but they do not spread to the medial surface of thighs; DD^∗^ = distinct darkening of the scrotal skin; HD^∗^ = hair distributions in adult both in quantity and type. They can spread to the medial surface of thigh and are described in the inverse triangle; SC^∗^ = the scrotums are of adult type with regard to shape and size.

## Supplementary Materials

Supplementary Materials Figure 1: scatter plot of plasma LH in different groups. Figures 2 and 3: plasma LH levels in Tanner stage I boys at different time points. Figures 4 and 5: plasma testosterone levels in Tanner stage I and II boys at different time points. Figures 6 and 7: plasma LH levels in Tanner stage II boys at different time points. Figures 8 and 9: plasma LH levels in Tanner stage III boys at different time points. Figures 10 and 11: plasma testosterone levels in Tanner stage III boys at different time points. Figures 12 and 13: plasma LH levels in Tanner stage IV boys at different time points. Figures 14 and 15: plasma testosterone levels in Tanner stage IV boys at different time points. Figures 16 and 17: plasma LH levels in Tanner stage V boys at different time points. Figures 18 and 19: plasma testosterone levels in Tanner stage V boys at different time points. Figures 20 and 21: plasma LH levels in adult men at different time points. Figures 22 and 23: plasma testosterone levels in adult men at different time points.

## References

[1] Teles M. G., Bianco S. D., Brito V. N. (2008). A *GPR54*-activating mutation in a patient with central precocious puberty. *The New England Journal of Medicine*.

[2] de Roux N., Genin E., Carel J. C., Matsuda F., Chaussain J. L., Milgrom E. (2003). Hypogonadotropic hypogonadism due to loss of function of the KiSS1-derived peptide receptor GPR54. *Proceedings of the National Academy of Sciences of the United States of America*.

[3] Seminara S. B., Messager S., Chatzidaki E. E. (2003). The *GPR54* gene as a regulator of puberty. *The New England Journal of Medicine*.

[4] Lanfranco F., Gromoll J., von Eckardstein S., Herding E. M., Nieschlag E., Simoni M. (2005). Role of sequence variations of the GnRH receptor and G protein-coupled receptor 54 gene in male idiopathic hypogonadotropic-hypogonadism. *European Journal of Endocrinology*.

[5] Semple R. K., Achermann J. C., Ellery J. (2005). Two novel missense mutations in g protein-coupled receptor 54 in a patient with hypogonadotropic hypogonadism. *The Journal of Clinical Endocrinology & Metabolism*.

[6] Cerrato F., Shagoury J., Kralickova M. (2006). Coding sequence analysis of *GNRHR* and *GPR54* in patients with congenital and adult-onset forms of hypogonadotropic hypogonadism. *European Journal of Endocrinology*.

[7] Tenenbaum-Rakover Y., Commenges-Ducos M., Iovane A., Aumas C., Admoni O., de Roux N. (2007). Neuroendocrine phenotype analysis in five patients with isolated hypogonadotropic hypogonadism due to a L102P inactivating mutation of GPR54. *The Journal of Clinical Endocrinology & Metabolism*.

[8] Nimri R., Lebenthal Y., Lazar L. (2011). A novel loss-of-function mutation in *GPR54/KISS1R* leads to hypogonadotropic hypogonadism in a highly consanguineous family. *The Journal of Clinical Endocrinology & Metabolism*.

[9] Funes S., Hedrick J. A., Vassileva G. (2003). The KiSS-1 receptor GPR54 is essential for the development of the murine reproductive system. *Biochemical and Biophysical Research Communications*.

[10] Lapatto R., Pallais J. C., Zhang D. (2007). Kiss1^−/−^ mice exhibit more variable hypogonadism than Gpr54^−/−^ mice. *Endocrinology*.

[11] Han S. K., Gottsch M. L., Lee K. J. (2005). Activation of gonadotropin-releasing hormone neurons by kisspeptin as a neuroendocrine switch for the onset of puberty. *The Journal of Neuroscience*.

[12] Tena-Sempere M. (2006). GPR54 and kisspeptin in reproduction. *Human Reproduction Update*.

[13] Rhie Y. J., Lee K. H., Eun S. H. (2011). Serum kisspeptin levels in Korean girls with central precocious puberty. *Journal of Korean Medical Science*.

[14] Demirbilek H., Gonc E. N., Ozon A., Alikasifoglu A., Kandemir N. (2012). Evaluation of serum kisspeptin levels in girls in the diagnosis of central precocious puberty and in the assessment of pubertal suppression. *Journal of Pediatric Endocrinology and Metabolism*.

[15] Clarkson J., Han S. K., Liu X., Lee K., Herbison A. E. (2010). Neurobiological mechanisms underlying kisspeptin activation of gonadotropin-releasing hormone (GnRH) neurons at puberty. *Molecular and Cellular Endocrinology*.

[16] Gill J. C., Wang O., Kakar S., Martinelli E., Carroll R. S., Kaiser U. B. (2010). Reproductive hormone-dependent and -independent contributions to developmental changes in kisspeptin in GnRH-deficient hypogonadal mice. *PLoS One*.

[17] De Bond J.-A. P., Li Q., Millar R. P., Clarke I. J., Smith J. T. (2013). Kisspeptin signaling is required for the luteinizing hormone response in anestrous ewes following the introduction of males. *PLoS One*.

[18] Brioude F., Bouligand J., Francou B. (2013). Two families with normosmic congenital hypogonadotropic hypogonadism and biallelic mutations in *KISS1R (KISS1 receptor)*: clinical evaluation and molecular characterization of a novel mutation. *PLoS One*.

[19] Skorupskaite K., George J. T., Anderson R. A. (2014). The kisspeptin-GnRH pathway in human reproductive health and disease. *Human Reproduction Update*.

[20] Terasawa E., Fernandez D. L. (2001). Neurobiological mechanisms of the onset of puberty in primates. *Endocrine Reviews*.

[21] Grumbach M. M. (2002). The neuroendocrinology of human puberty revisited. *Hormone Research in Paediatrics*.

[22] Plant T. M., Barker-Gibb M. L. (2004). Neurobiological mechanisms of puberty in higher primates. *Human Reproduction Update*.

[23] Plant T. M. (2001). Neurobiological bases underlying the control of the onset of puberty in the rhesus monkey: a representative higher primate. *Frontiers in Neuroendocrinology*.

[24] El-Majdoubi M., Sahu A., Ramaswamy S., Plant T. M. (2000). Neuropeptide Y: a hypothalamic brake restraining the onset of puberty in primates. *Proceedings of the National Academy of Sciences of the United States of America*.

[25] Terasawa E., Kurian J. R., Keen K. L., Shiel N. A., Colman R. J., Capuano S. V. (2012). Body weight impact on puberty: effects of high-calorie diet on puberty onset in female rhesus monkeys. *Endocrinology*.

[26] Plant T. M., Fraser M. O., Medhamurthy R., Gay V. L., Delamarre-van de Waal H. A., Plant T. M., Rees G. P., Schoemaker J. (1989). Somatogenic control of GnRH neuronal synchronization during development in primates: a speculation. *Control of the Onset of Puberty III*.

[27] Wilson M. E., Gordon T. P., Rudman C. G., Tanner J. M. (1989). Effects of growth hormone on the tempo of sexual maturation in female rhesus monkeys. *The Journal of Clinical Endocrinology & Metabolism*.

[28] Mann D. R., Plant T. M. (2010). The role and potential sites of action of thyroid hormone in timing the onset of puberty in male primates. *Brain Research*.

[29] Marshall W. A., Tanner J. M. (1969). Variations in pattern of pubertal changes in girls. *Archives of Disease in Childhood*.

[30] Bond J. P. D., Smith J. T. (2014). Kisspeptin and energy balance in reproduction. *Reproduction*.

[31] Pankov A. Y. (2015). Kisspeptin and leptin in the regulation of fertility. *Molecular Biology*.

[32] Dhillo W. S., Chaudhri O. B., Patterson M. (2005). Kisspeptin-54 stimulates the hypothalamic-pituitary gonadal axis in human males. *The Journal of Clinical Endocrinology & Metabolism*.

[33] Navarro V. M., Castellano J. M., Fernandez-Fernandez R. (2004). Developmental and hormonally regulated messenger ribonucleic acid expression of KiSS-1 and its putative receptor, GPR54, in rat hypothalamus and potent luteinizing hormone-releasing activity of KiSS-1 peptide. *Endocrinology*.

[34] Shahab M., Mastronardi C., Seminara S. B., Crowley W. F., Ojeda S. R., Plant T. M. (2005). Increased hypothalamic GPR54 signaling: a potential mechanism for initiation of puberty in primates. *Proceedings of the National Academy of Sciences of the United States of America*.

[35] de Vries L., Shtaif B., Phillip M., Gat-Yablonski G. (2009). Kisspeptin serum levels in girls with central precocious puberty. *Clinical Endocrinology*.

[36] Feingold D. (1992). Atlas of physical diagnosis. *Pediatric Endocrinology*.

[37] Holford N. H. (1996). A size standard for pharmacokinetics. *Clinical Pharmacokinetics*.

[38] George J. T., Veldhuis J. D., Roseweir A. K. (2011). Kisspeptin-10 is a potent stimulator of LH and increases pulse frequency in men. *The Journal of Clinical Endocrinology & Metabolism*.

[39] Semaan S. J., Kauffman A. S. (2015). Daily successive changes in reproductive gene expression and neuronal activation in the brains of pubertal female mice. *Molecular and Cellular Endocrinology*.

[40] Mitsushima D., Hei D. L., Terasawa E. (1994). Gamma-aminobutyric acid is an inhibitory neurotransmitter restricting the release of luteinizing hormone-releasing hormone before the onset of puberty. *Proceedings of the National Academy of Sciences of the United States of America*.

[41] Keen K. L., Burich A. J., Mitsushima D., Kasuya E., Terasawa E. (1999). Effects of pulsatile infusion of the GABAA receptor blocker bicuculline on the onset of puberty in female rhesus monkeys. *Endocrinology*.

[42] Nakada K., Ishikawa Y., Nakao T., Sawamukai Y. (2002). Changes in responses to GnRH on luteinizing hormone and follicle stimulating hormone secretion in prepubertal heifers. *Journal of Reproduction and Development*.

[43] Kotani M., Detheux M., Vandenbogaerde A. (2001). The metastasis suppressor gene KiSS-1 encodes kisspeptins, the natural ligands of the orphan G protein-coupled receptor GPR54. *Journal of Biological Chemistry*.

[44] Ramaswamy S., Seminara S. B., Pohl C. R. D., Pietro M. J., WFJr C., Plant T. M. (2007). Effect of continuous intravenous administration of human metastin 45–54 on the neuroendocrine activity of the hypothalamic-pituitary-testicular axis in the adult male rhesus monkey (*Macaca mulatta*). *Endocrinology*.

[45] Anjum S., Krishna A., Sridaran R., Tsutsui K. (2012). Localization of gonadotropin-releasing hormone (GnRH), gonadotropin-inhibitory hormone (GnIH), kisspeptin and GnRH receptor and their possible roles in testicular activities from birth to senescence in mice. *Journal of Experimental Zoology Part A: Ecological Genetics and Physiology*.

[46] Irfan S., Ehmcke J., Wahab F., Shahab M., Schlatt S. (2013). Intratesticular action of kisspeptin in rhesus monkey (*Macaca mulatta*). *Andrologia*.

[47] Mann D. R., Johnoson A. K., Gimple T., Castracane V. D. (2003). Changes in circulating leptin, leptin receptor, and gonadal hormones from infancy until advanced age in humans. *The Journal of Clinical Endocrinology & Metabolism*.

[48] Ioannis E. M., Spyros D. M. (1999). Leptin in human reproduction. *Human Reproduction Update*.

[49] Smith J. T., Cunningham M. J., Rissman E. F., Clifton D. F., Steiner R. A. (2005). Regulation of *Kiss1* gene expression in the brain of the female mouse. *Endocrinology*.

[50] Cioffi J. A., Shafer A. W., Zupancic T. J. (1996). Novel B219/OB receptor isoforms: possible role of leptin in hematopoiesis and reproduction. *Nature Medicine*.

[51] Moschos S., Chan J. L., Mantzoros C. S. (2002). Leptin and reproduction: a review. *Fertility and Sterility*.

[52] Bellver J., Melo M. A., Bosch E., Serra V., Remohi J., Pellicer A. (2007). Obesity and poor reproductive outcome: the potential role of the endometrium. *Fertility and Sterility*.

[53] Luque R. M., Kineman R. D., Tena-Sempere M. (2007). Regulation of hypothalamic expression of KiSS-1 and GPR54 genes by metabolic factors: analyses using mouse models and a cell line. *Endocrinology*.

[54] Backholer K., Smith J. T., Rao A. (2010). Kisspeptin cells in the ewe brain respond to leptin and communicate with neuropeptide Y and proopiomelanocortin cells. *Endocrinology*.

[55] Morelli A., Marini M., Mancina R. (2008). Sex steroids and leptin regulate the “first Kiss” (KiSS 1/G-protein-coupled receptor 54 system) in human gonadotropin-releasing-hormone-secreting neuroblasts. *The Journal of Sexual Medicine*.

[56] Palmert M. R., Radovick S., Boepple P. A. (1998). Leptin levels in children with central precocious puberty. *The Journal of Clinical Endocrinology & Metabolism*.

[57] Terasawa E., Guerriero K. A., Plant T. M., Kauffman A. S., Smith J. T. (2013). Kisspeptin and puberty in mammals. *Kisspeptin Signaling in Reproductive Biology*.

[58] Burr I. M., Izonenko P. C., Kaplan S. L., Grumbach M. M. (1970). Hormonal changes in puberty I. Correlation of serum luteinizing hormone and follicle stimulating hormone with stages of puberty, testicular size, and bone age in normal boys. *Pediatric Research*.

[59] Albertson-Wikland K., Rosberg S., Lannering B. (1997). Twenty-four-hour profiles of luteinizing hormone, follicle-stimulating hormone, testosterone, and estradiol levels: a semilongitudinal study throughout puberty in healthy boys. *The Journal of Clinical Endocrinology & Metabolism*.

[60] Jayasena C. N., Nijher G. M., Chaudhri O. B. (2009). Subcutaneous injection of kisspeptin-54 acutely stimulates gonadotropin secretion in women with hypothalamic amenorrhea, but chronic administration causes tachyphylaxis. *The Journal of Clinical Endocrinology & Metabolism*.

[61] Irwig M. S., Fraley G. S., Smith J. T. (2004). Kisspeptin activation of gonadotropin releasing hormone neurons and regulation of KiSS-1 mRNA in the male rat. *Neuro-endocrinology*.

[62] Carabulea G., Bughi S., Klepsch I., Eşanu C. (1980). Circulating FSH, LH, GH, testosterone, TSH, T3, T4, prolactin and insulin in boys during puberty. *Endocrinologie*.

