# Supplementary material for: Changes in the Responsiveness of the Hypothalamic-Pituitary-Gonadal Axis to Kisspeptin-10 Administration during Pubertal Transition in Boys
Source: Int J Endocrinol. 2018 Jun 26;2018:1475967. doi: 10.1155/2018/1475967 (PMC6038494; doi:10.1155/2018/1475967)
Supplement: Supplementary Materials — Figure 1: scatter plot of plasma LH in different groups. Figures 2 and 3: plasma LH levels in Tanner stage I boys at different time points. Figures 4 and 5: plasma testosterone levels in Tanner stage I and II boys at different time points. Figures 6 and 7: plasma LH levels in Tanner stage II boys at different time points. Figures 8 and 9: plasma LH levels in Tanner stage III boys at different time points. Figures 10 and 11: plasma testosterone levels in Tanner stage III boys at different time points. Figures 12 and 13: plasma LH levels in Tanner stage IV boys at different time points. Figures 14 and 15: plasma testosterone levels in Tanner stage IV boys at different time points. Figures 16 and 17: plasma LH levels in Tanner stage V boys at different time points. Figures 18 and 19: plasma testosterone levels in Tanner stage V boys at different time points. Figures 20 and 21: plasma LH levels in adult men at different time points. Figures 22 and 23: plasma testosterone levels in adult men at different time points. [file 1475967.f1.pdf]

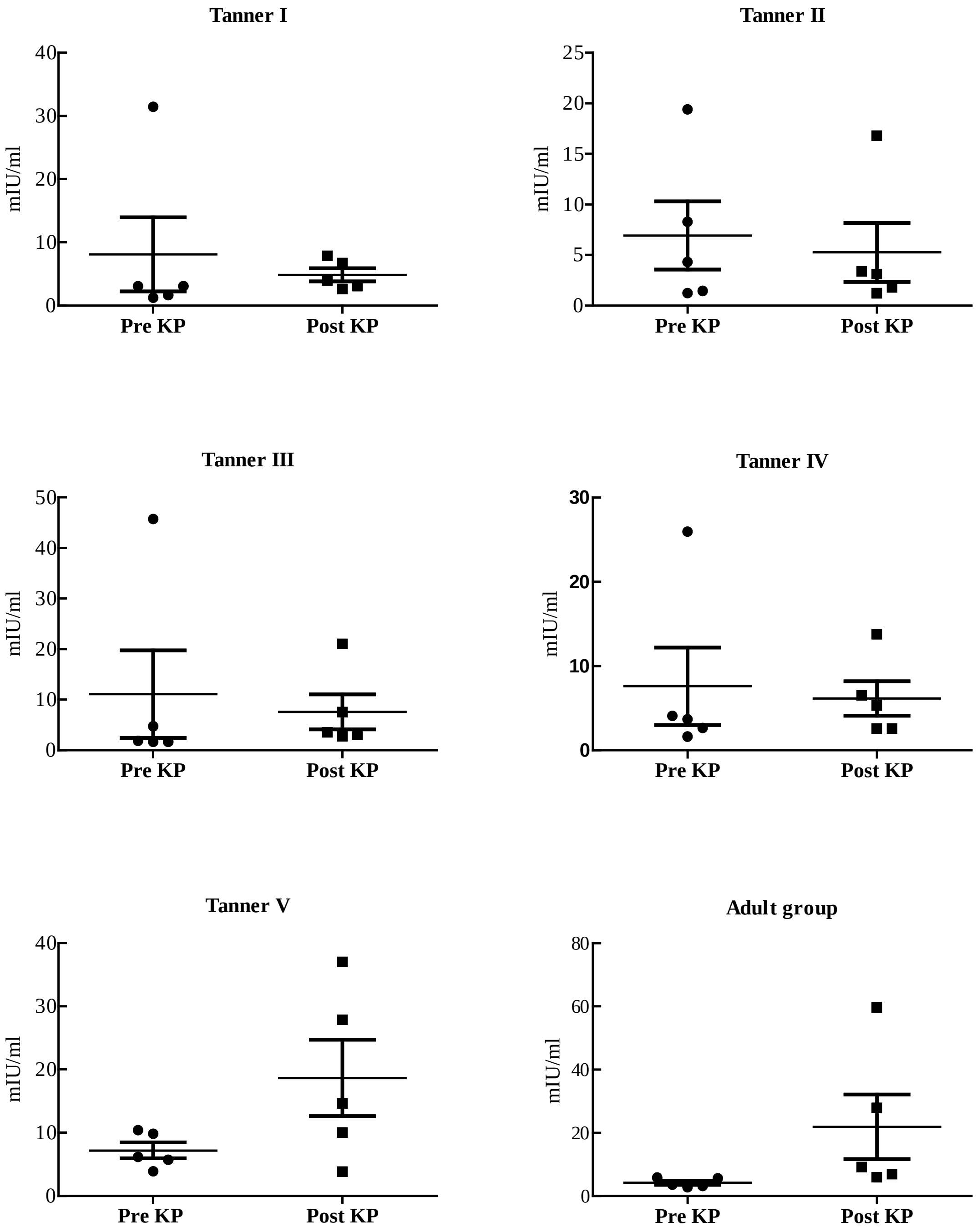

Figure 1: Scatter plot of plasma LH in different groups

Plasma LH levels in Tanner stage I boys at different time points

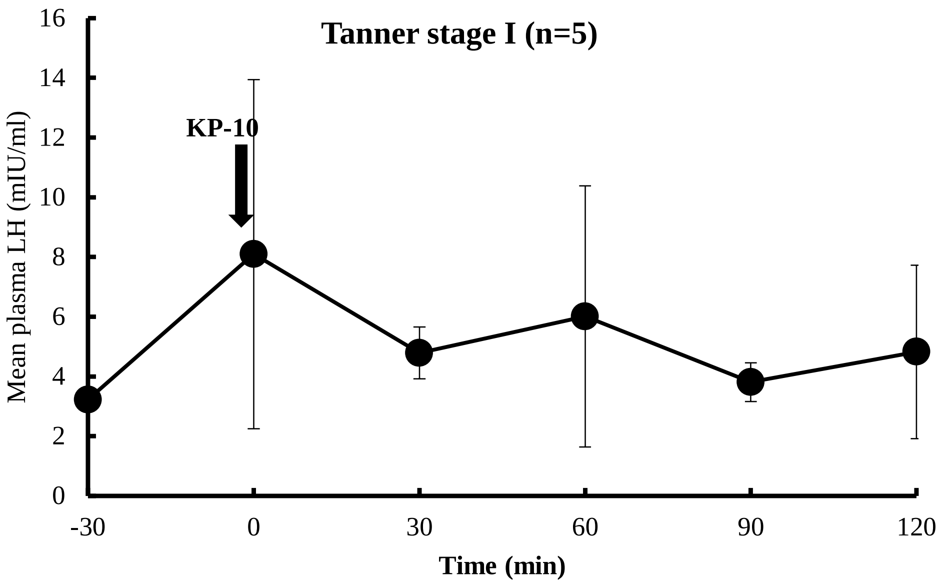

Figure 2: Mean±SEM (n=5) plasma LH concentrations before and after administration of kisspeptin-10 (single iv bolus; ↓) in Tanner stage I boys. One–way ANOVA with repeated measures showed that there is no significant variation in plasma LH concentrations before and after kisspeptin administrations.

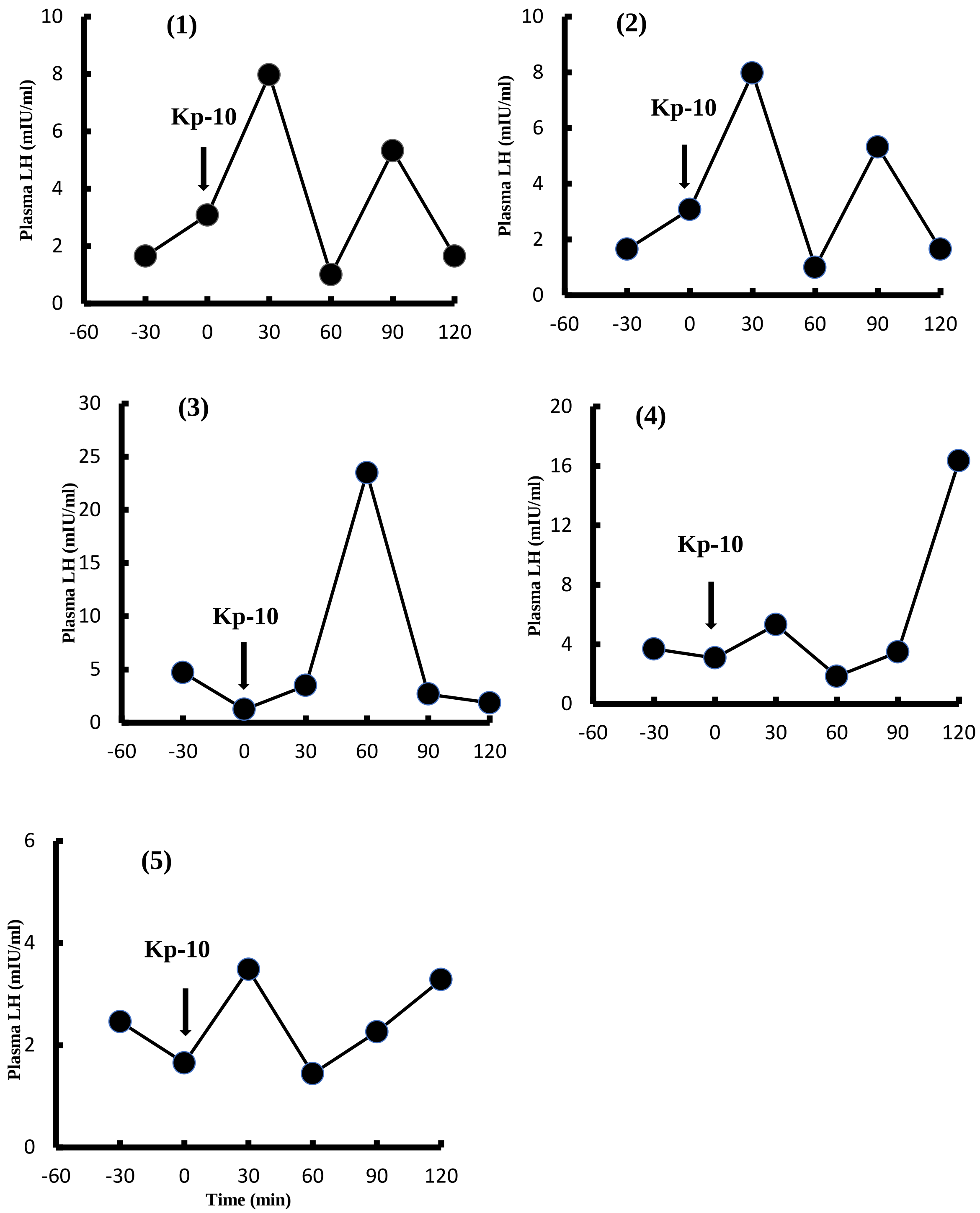

Figure 3: Plasma LH concentration before and after administration of kisspeptin-10 (single iv bolus; ↓) in individual Tanner stage I group (1-5).

Plasma Testosterone levels in Tanner stage I and II boys at different time points

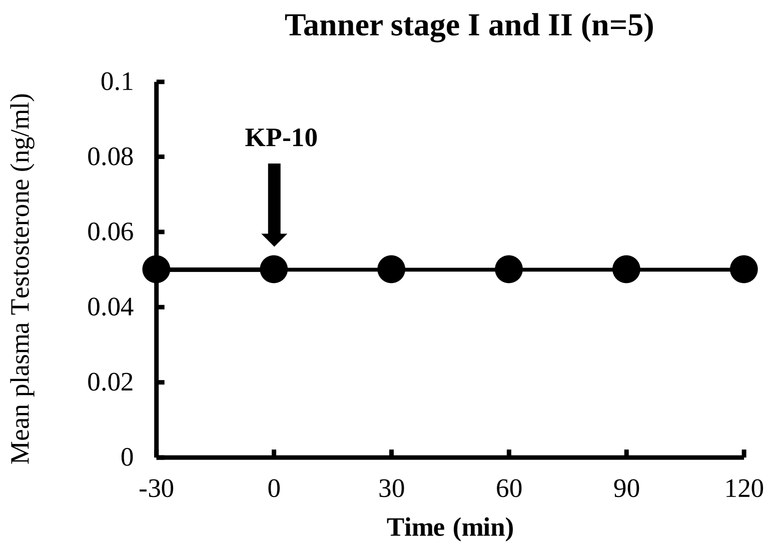

Figure 4: Mean±SEM (n=5) plasma testosterone concentrations before and after administration of kisspeptin-10 (single iv bolus; ↓) in Tanner stage I and II boys. One–way ANOVA with repeated measures showed that there is no significant variation in plasma testosterone concentrations after kisspeptin administration.

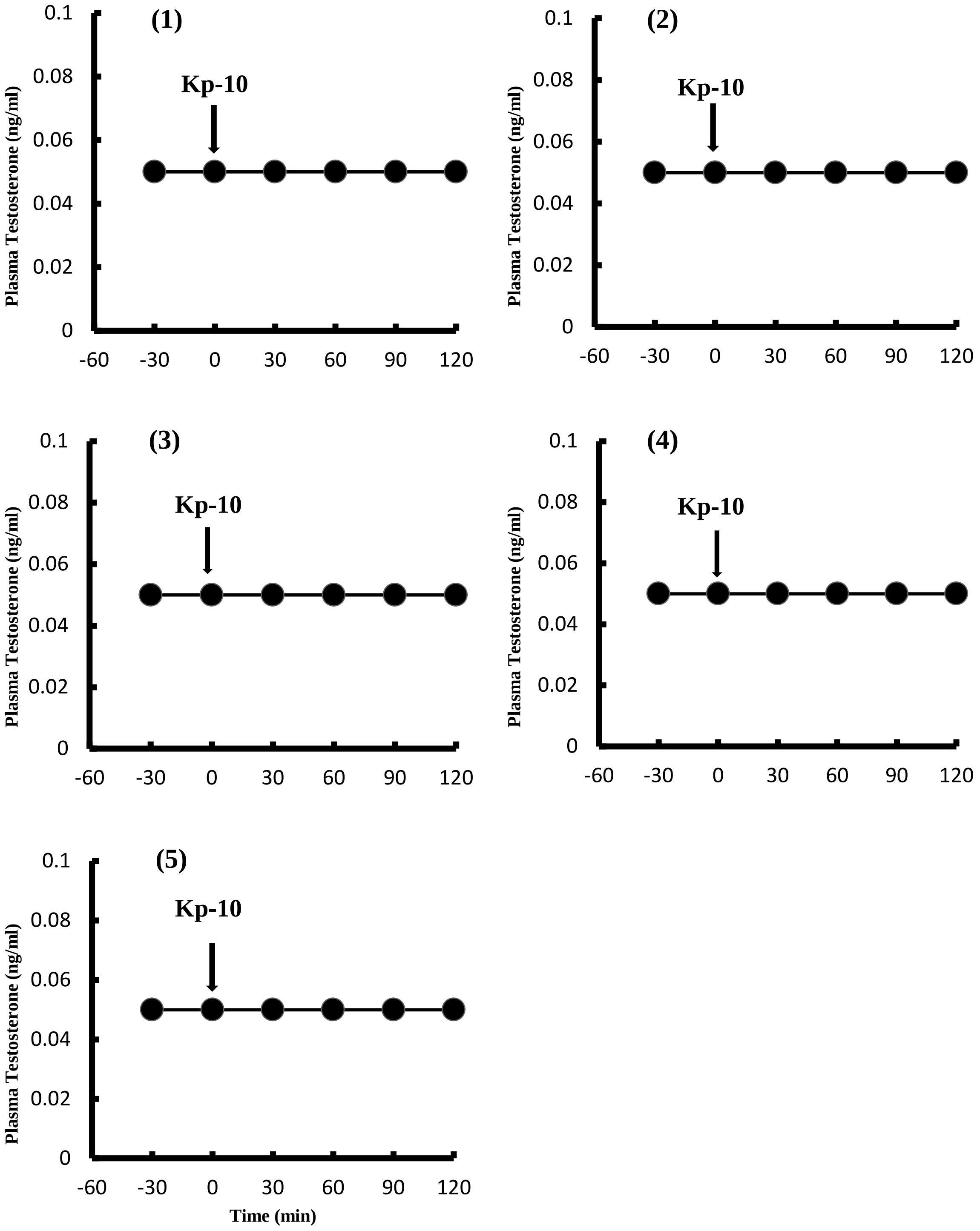

Figure 5: Plasma testosterone concentration before and after administration of kisspeptin-10 (single iv bolus; ↓) in individual Tanner stage I and II groups (1-5).

Plasma LH levels in Tanner stage II boys at different time points

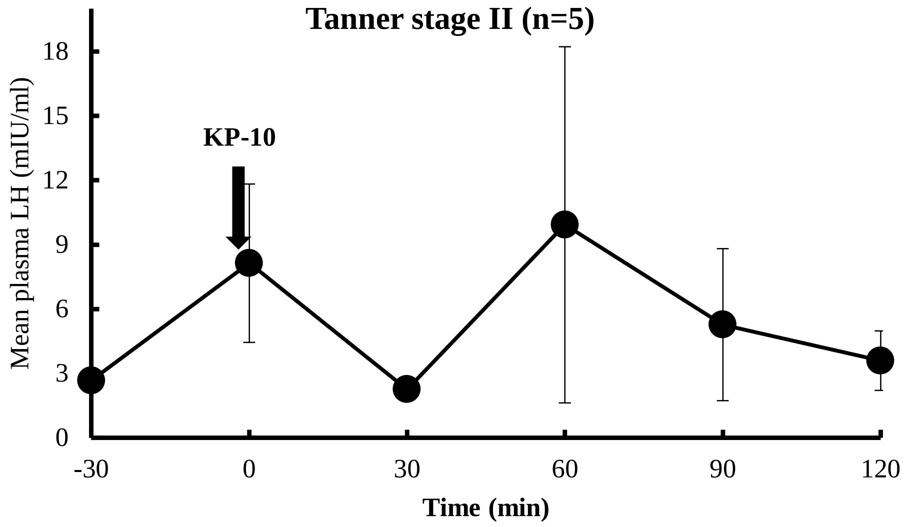

Figure 6: Mean±SEM (n=5) plasma LH concentrations before and after administration of kisspeptin-10 (single iv bolus; ↓) in Tanner stage II boys. One–way ANOVA with repeated measures showed that there is no significant variation in plasma LH concentrations before and after kisspeptin administrations.

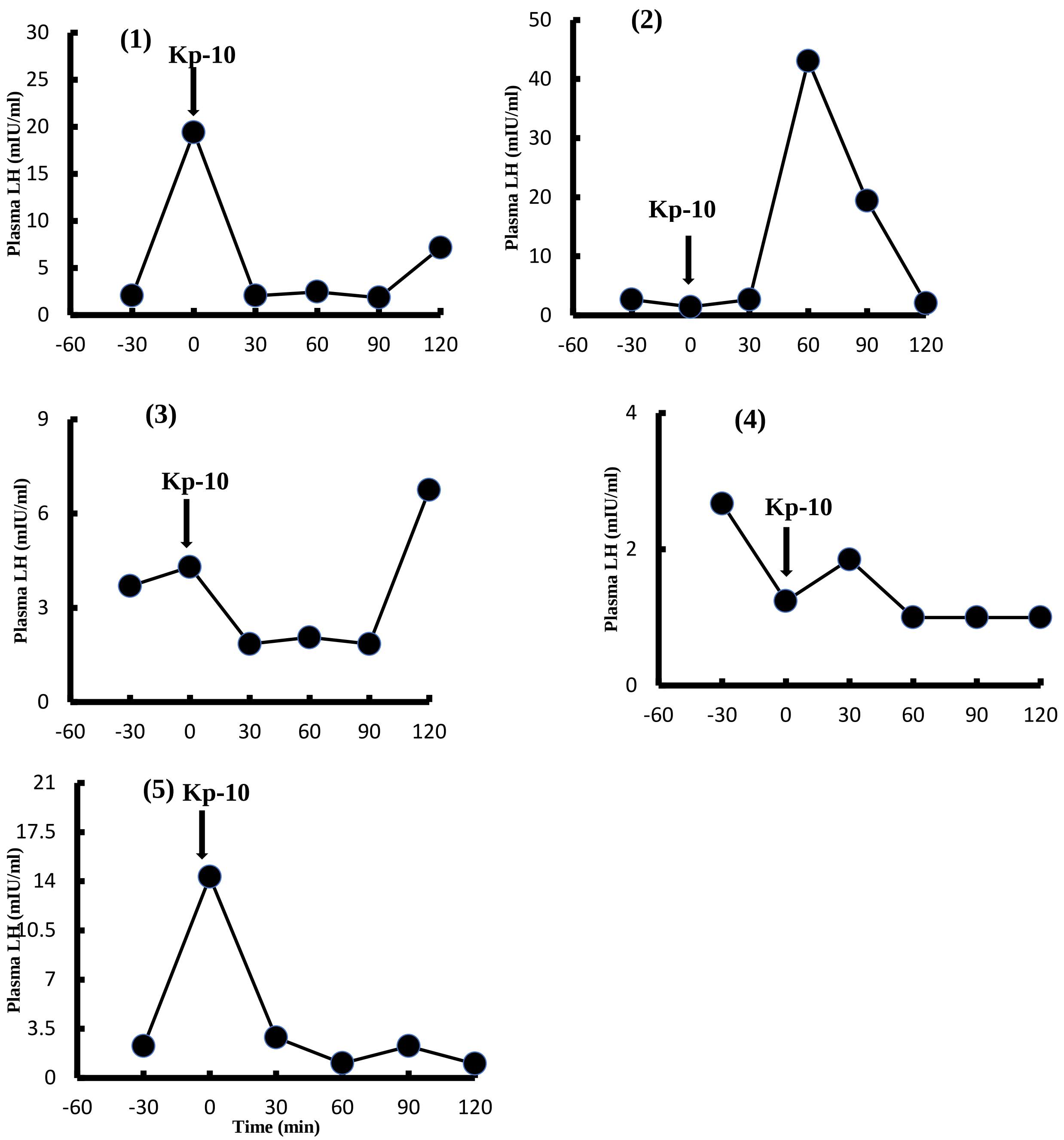

Figure 7: Plasma LH concentration before and after administration of kisspeptin-10 (single iv bolus; ↓) in individual Tanner stage II group (1-5).

Plasma LH levels in Tanner stage III boys at different time points

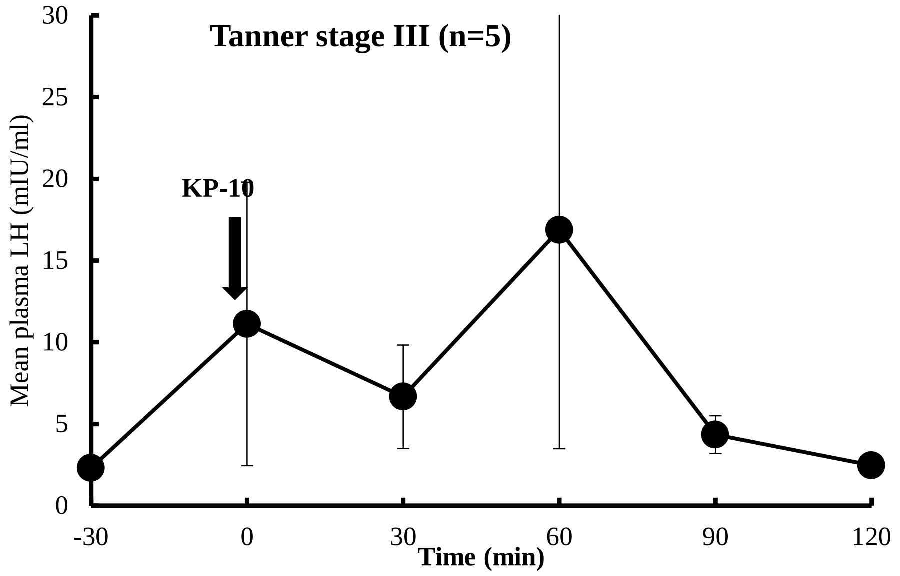

Figure 8: Mean±SEM (n=5) plasma LH concentrations before and after administration of kisspeptin-10 (single iv bolus; ↓) in Tanner stage III boys. One–way ANOVA with repeated measures showed that there is no significant variation in plasma LH concentrations before and after kisspeptin administrations.

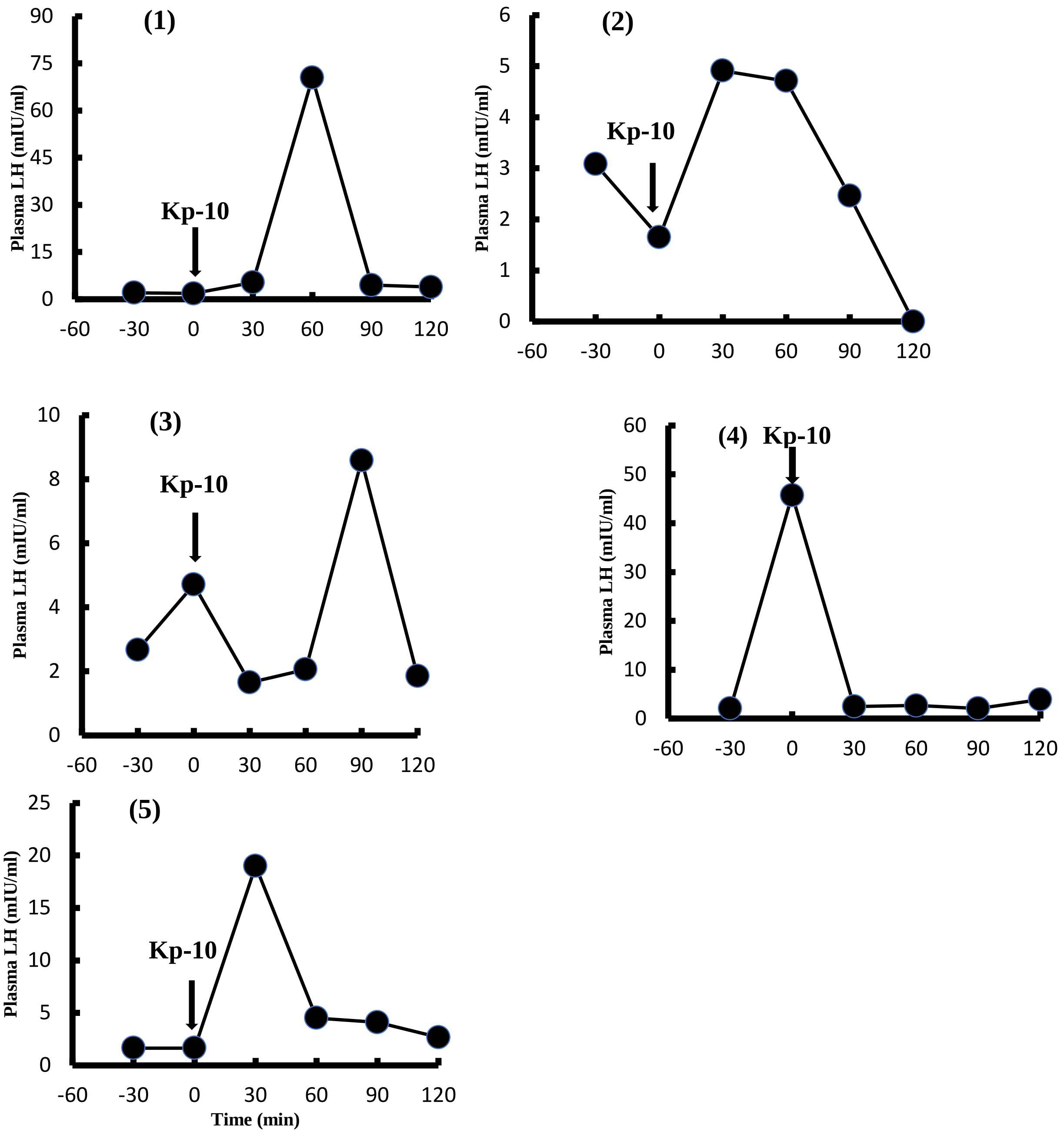

Figure 9: Plasma LH concentration before and after administration of kisspeptin-10 (single iv bolus; ↓) in individual Tanner stage III group (1-5).

Plasma Testosterone levels in Tanner stage III boys at different time points

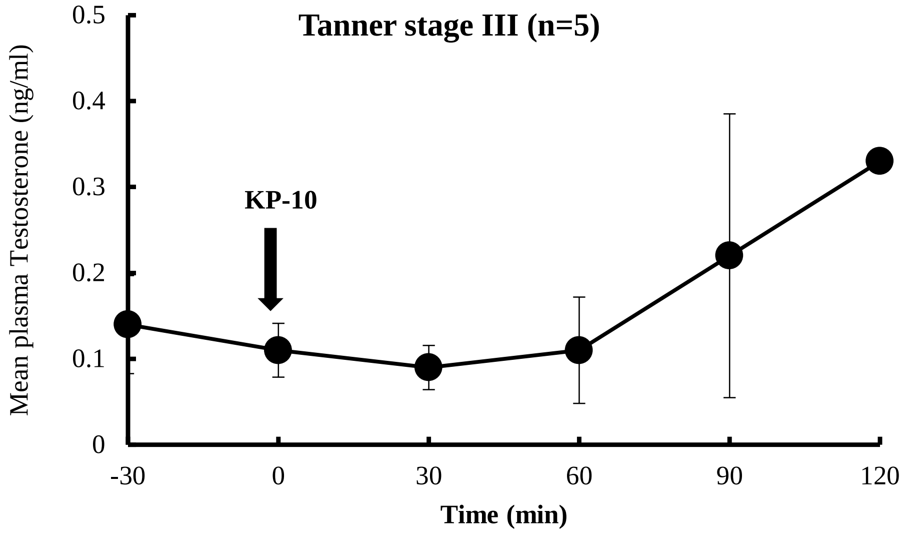

Figure 10: Mean±SEM (n=5) plasma testosterone concentrations before and after administration of kisspeptin-10 (single iv bolus; ↓) in Tanner stage III boys. One–way ANOVA with repeated measures showed that there is no significant variation in plasma testosterone concentrations after kisspeptin administrations.

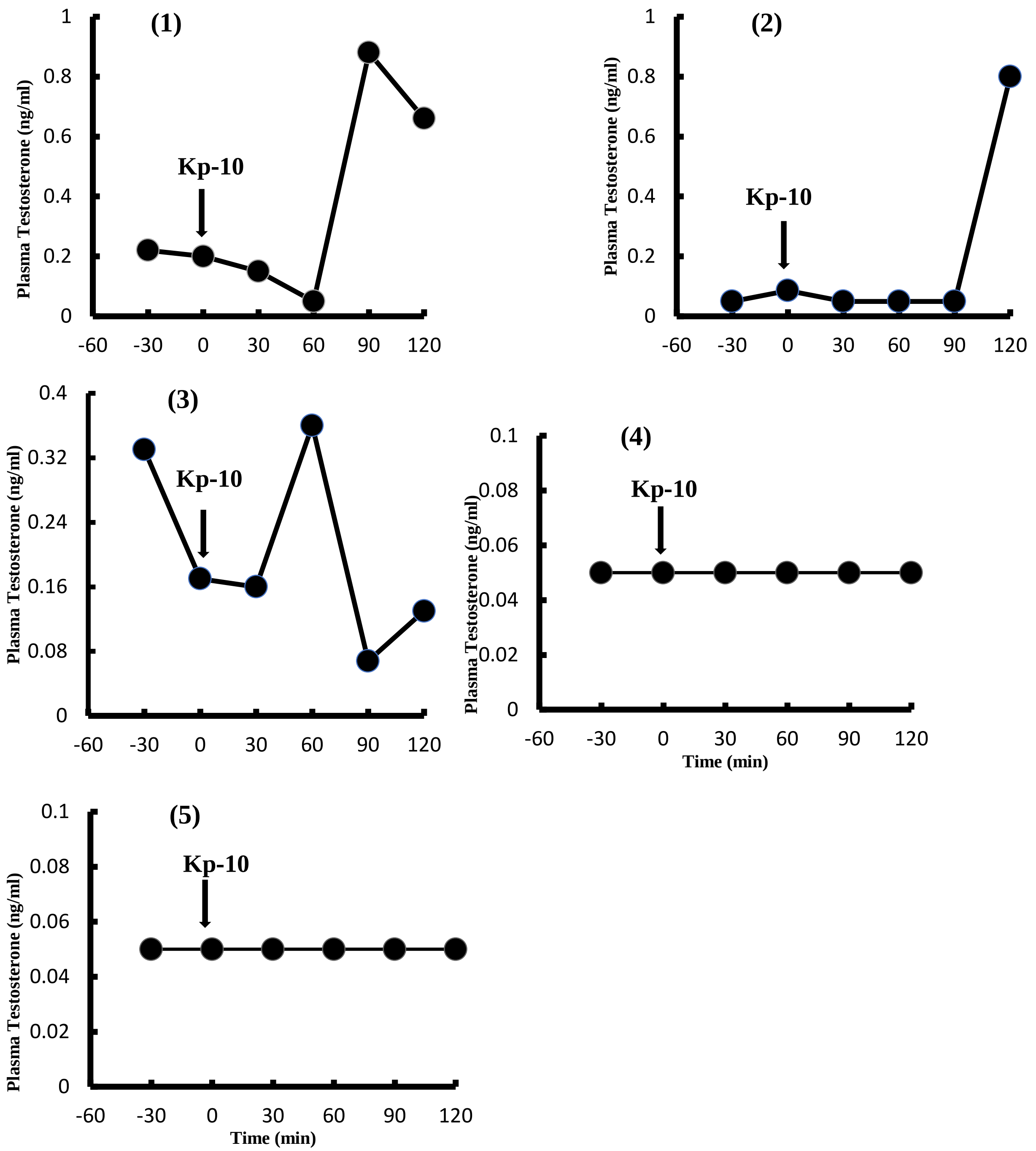

Figure 11: Plasma testosterone concentration before and after administration of kisspeptin-10 (single iv bolus; ↓) in individual Tanner stage III group (1-5).

Plasma LH levels in Tanner stage IV boys at different time points

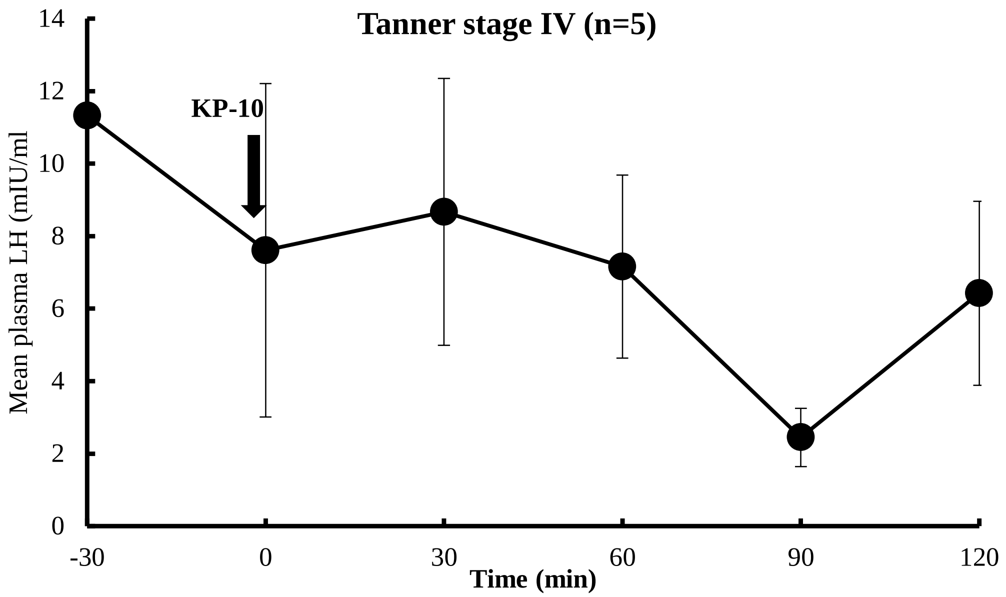

Figure 12: Mean ±SEM (n=5) plasma LH concentrations before and after administration of kisspeptin-10 (single iv bolus; ↓) in Tanner stage IV boys. One–way ANOVA with repeated measures showed that there is no significant variation in plasma LH concentrations before and after kisspeptin administrations.

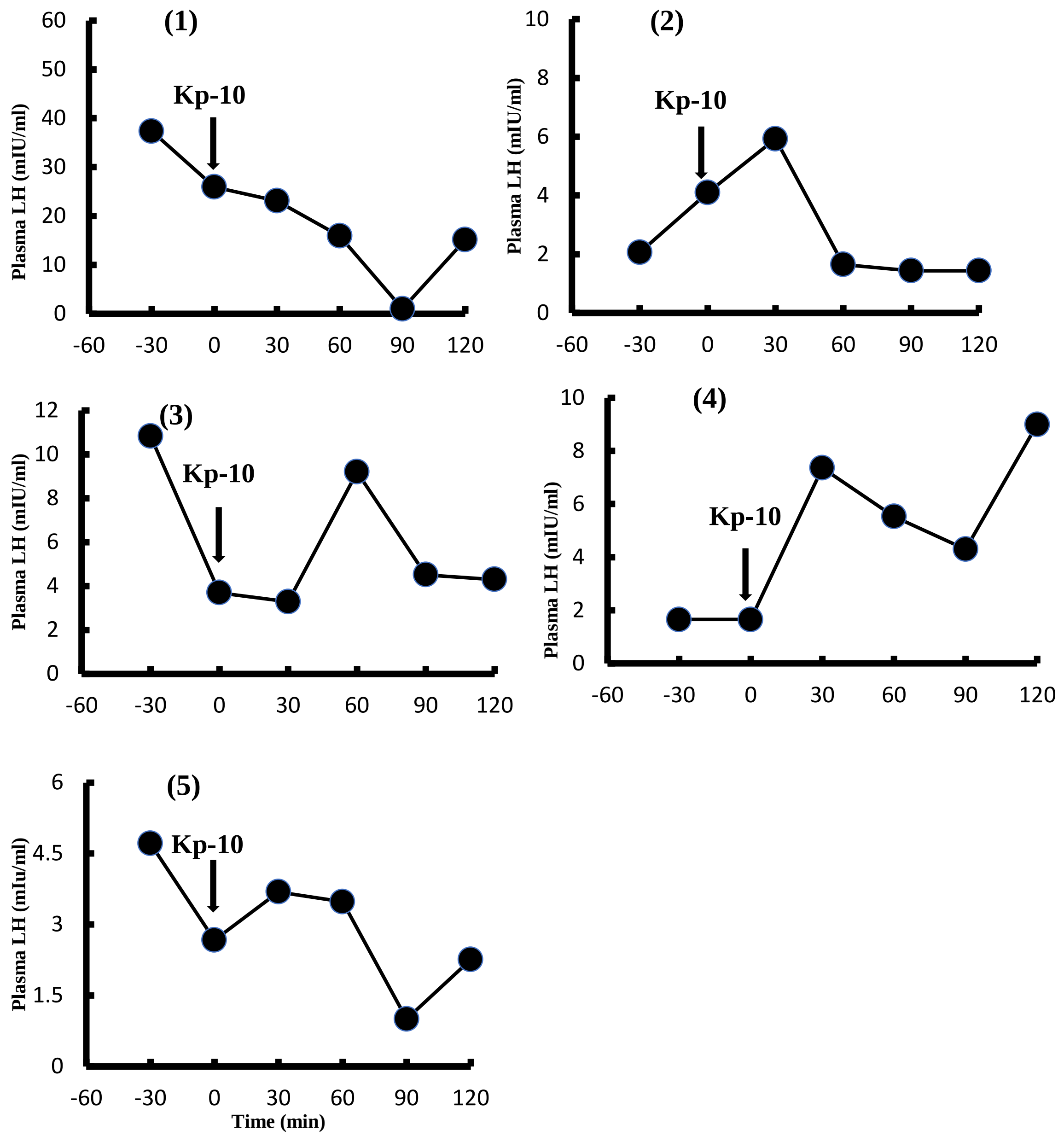

Figure 13: Plasma LH concentration before and after administration of kisspeptin-10 (single iv bolus; ↓) in individual Tanner stage IV group (1-5).

Plasma Testosterone levels in Tanner stage IV boys at different time points

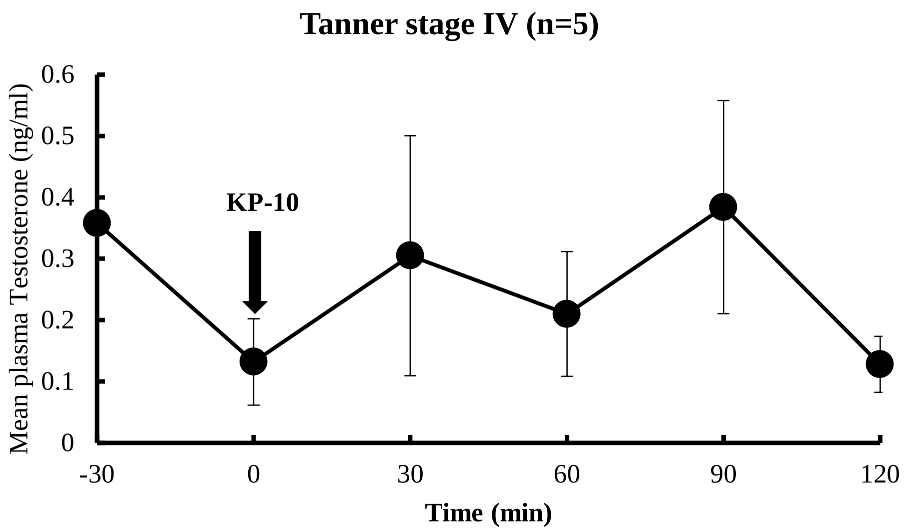

Figure 14: Mean±SEM (n=5) plasma testosterone concentrations before and after administration of kisspeptin-10 (single iv bolus; ↓) in Tanner stage IV boys. One–way ANOVA with repeated measures showed that there is no significant variation in plasma testosterone concentrations after kisspeptin administration.

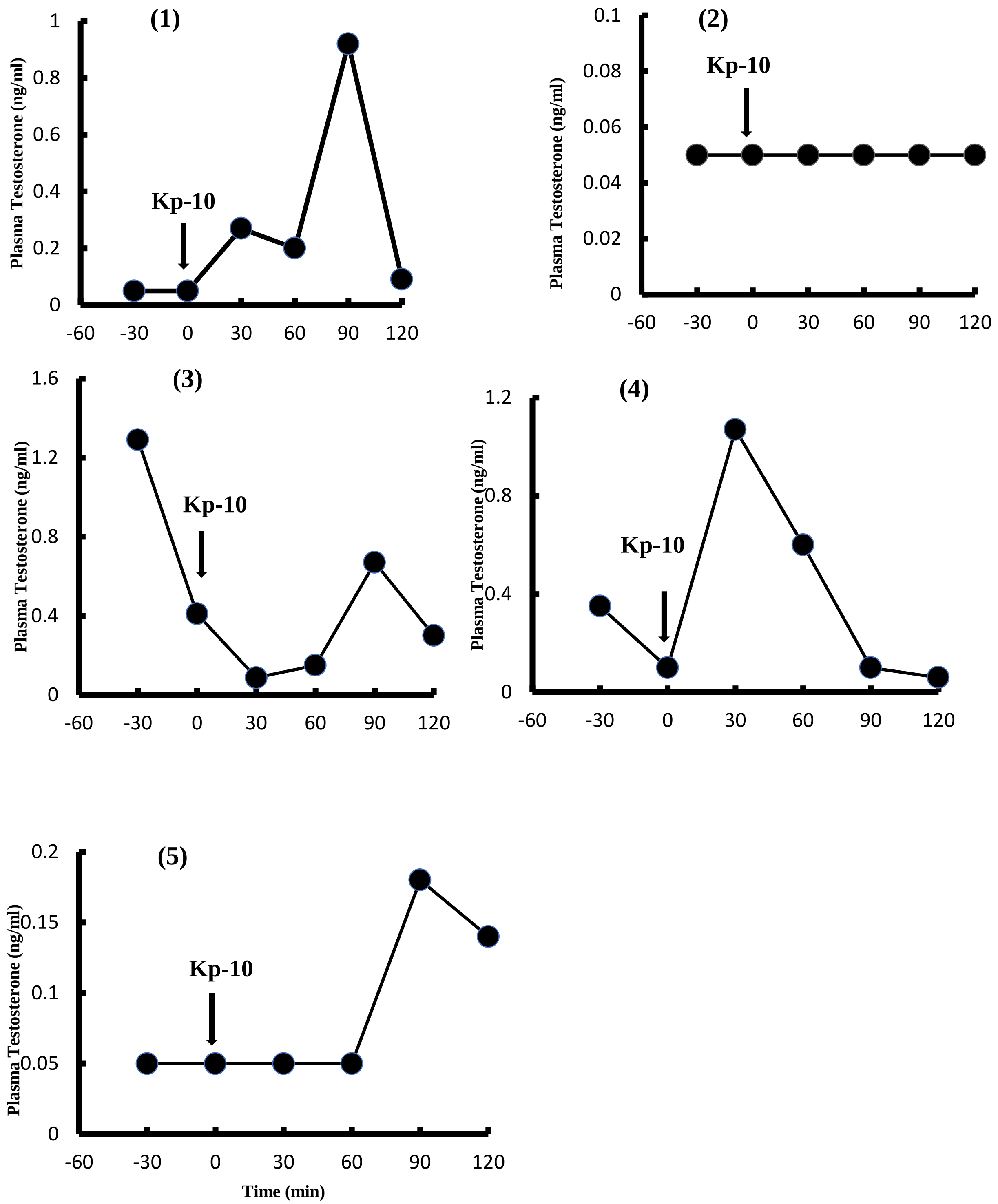

Figure 15: Plasma testosterone concentration before and after administration of kisspeptin-10 (single iv bolus; ↓) in individual Tanner stage IV group (1-5).

Plasma LH levels in Tanner stage V boys at different time points

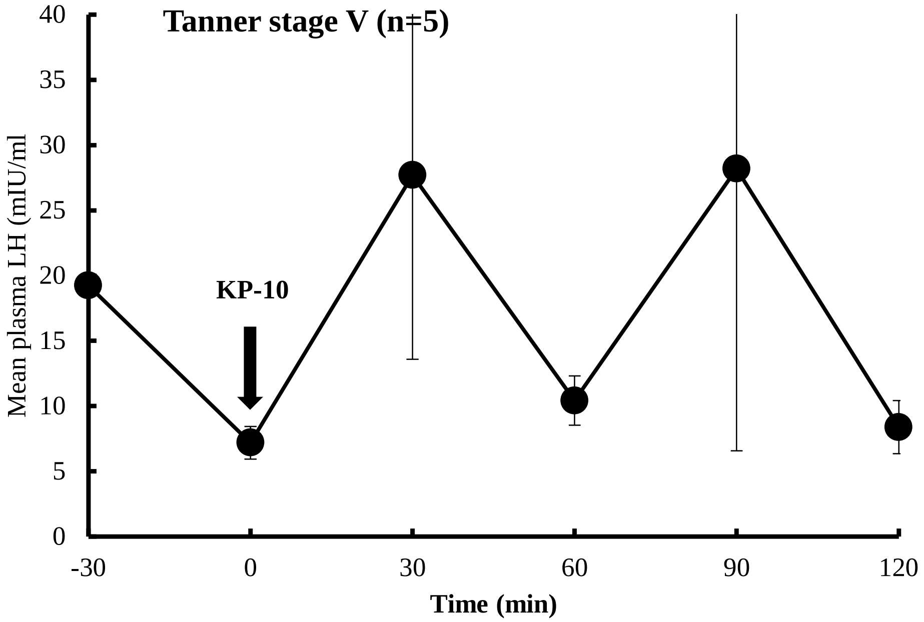

Figure 16: Mean±SEM (n=5) plasma LH concentrations before and after administration of kisspeptin-10 (single iv bolus; ↓) in Tanner stage V boys. One–way ANOVA with repeated measures showed that there is no significant variation in plasma LH concentrations before and after kisspeptin administrations.

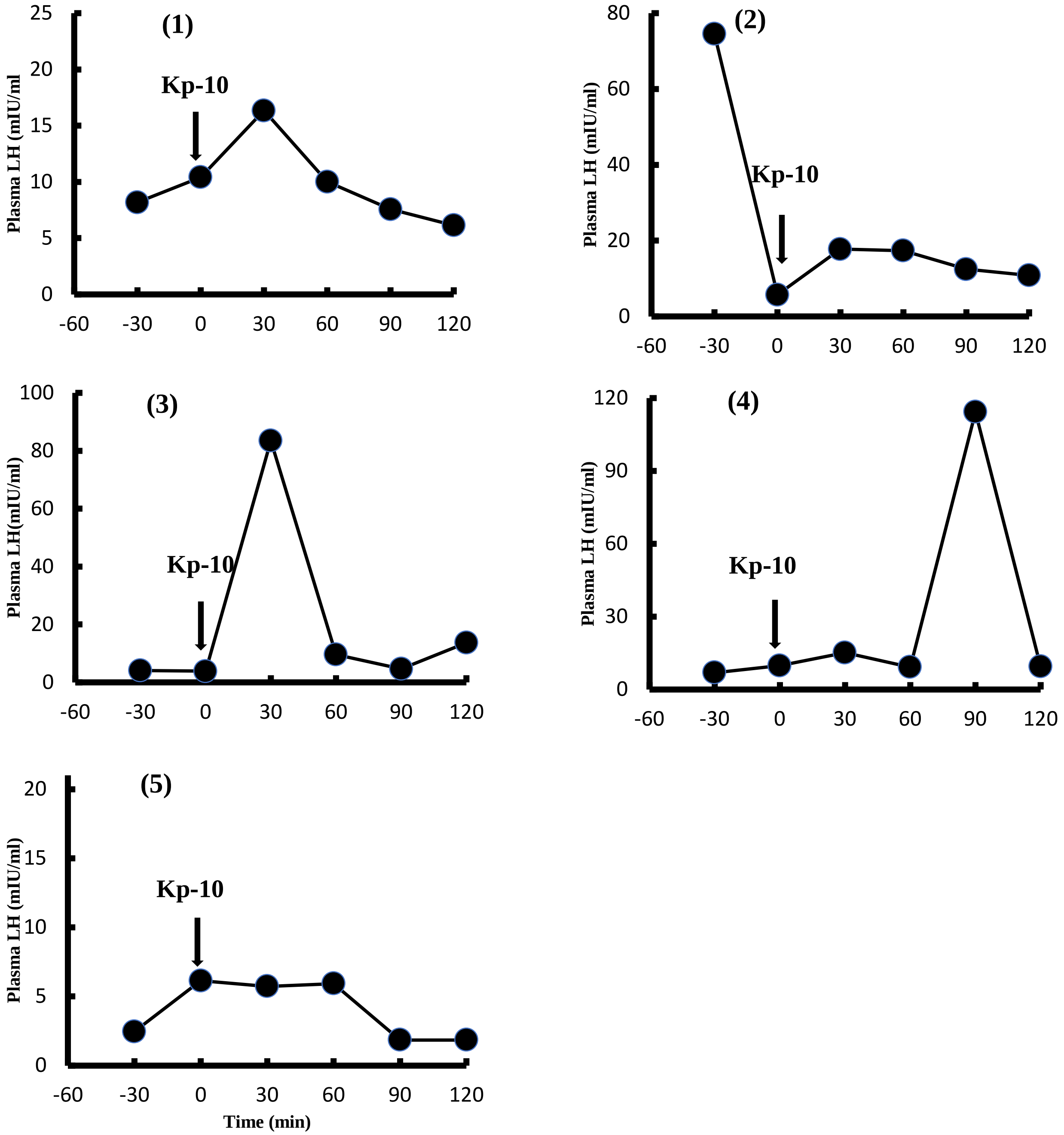

Figure 17: Plasma LH concentration before and after administration of kisspeptin-10 (single iv bolus; ↓) in individual Tanner stage V group (1-5).

Plasma Testosterone levels in Tanner stage V boys at different time points

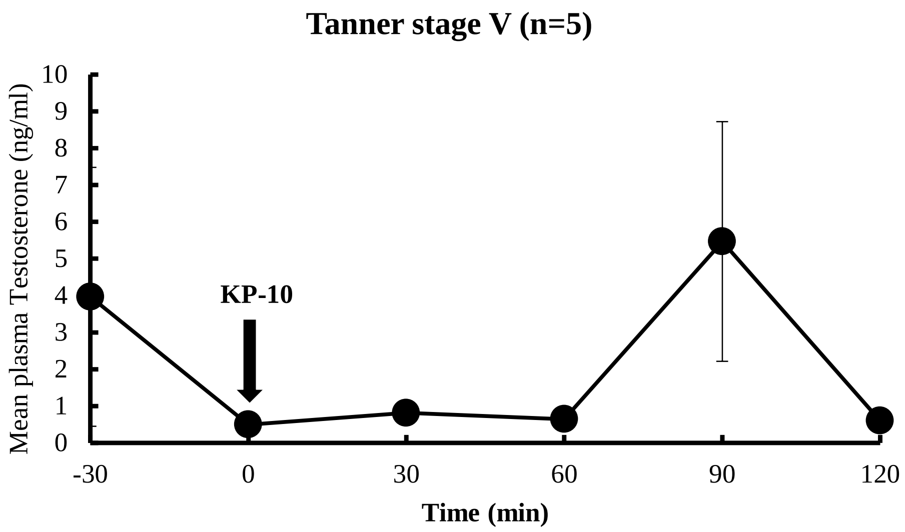

Figure 18: Mean±SEM (n=5) plasma testosterone concentrations before and after administration of kisspeptin-10 (single iv bolus; ↓) in Tanner stage V boys. One–way ANOVA with repeated measures showed that there is no significant variation in plasma testosterone concentrations after kisspeptin administrations.

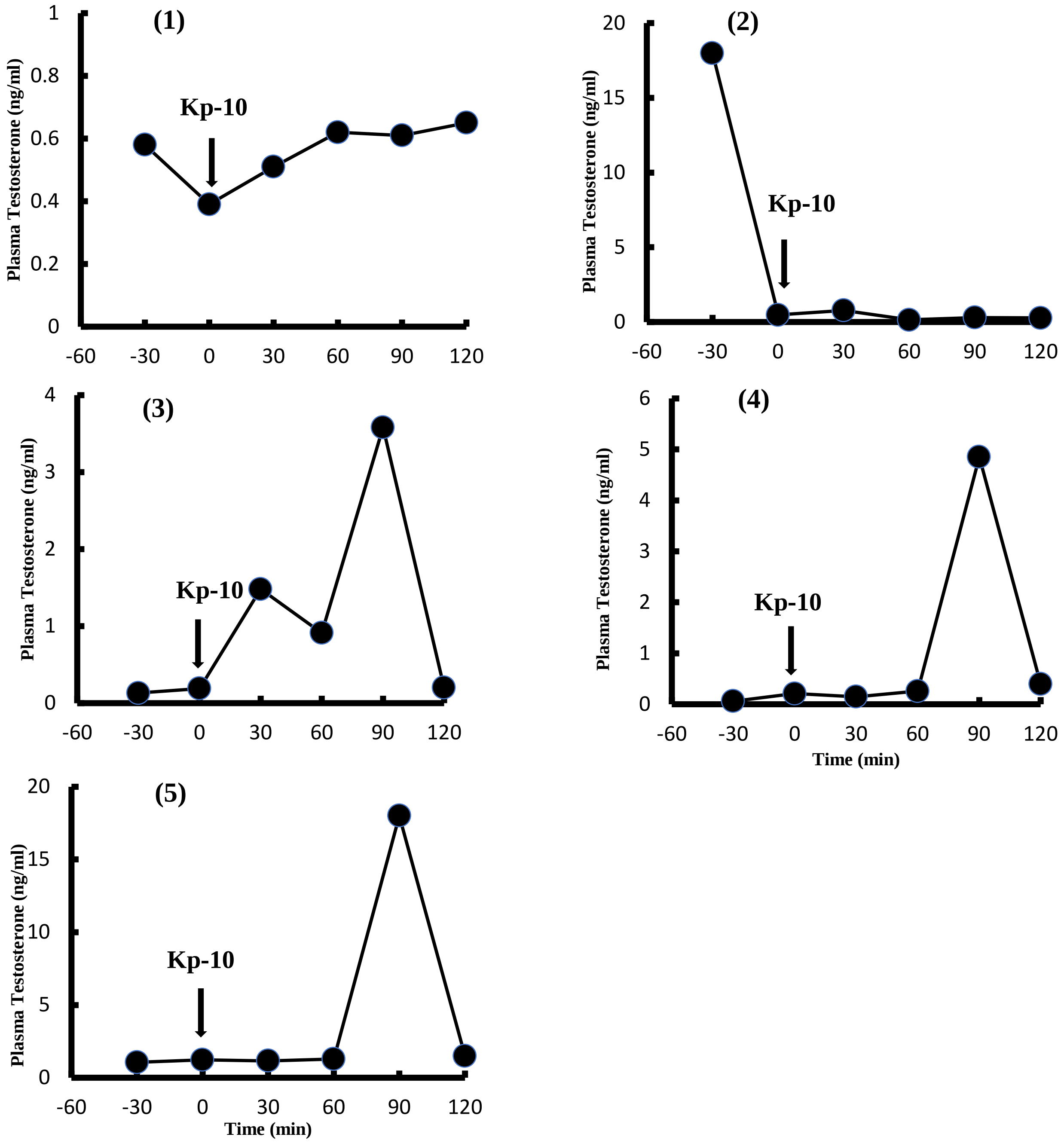

Figure 19: Plasma testosterone concentration before and after administration of kisspeptin-10 (single iv bolus; ↓) in individual Tanner stage V group (1-5).

Plasma LH levels in adult men at different time points

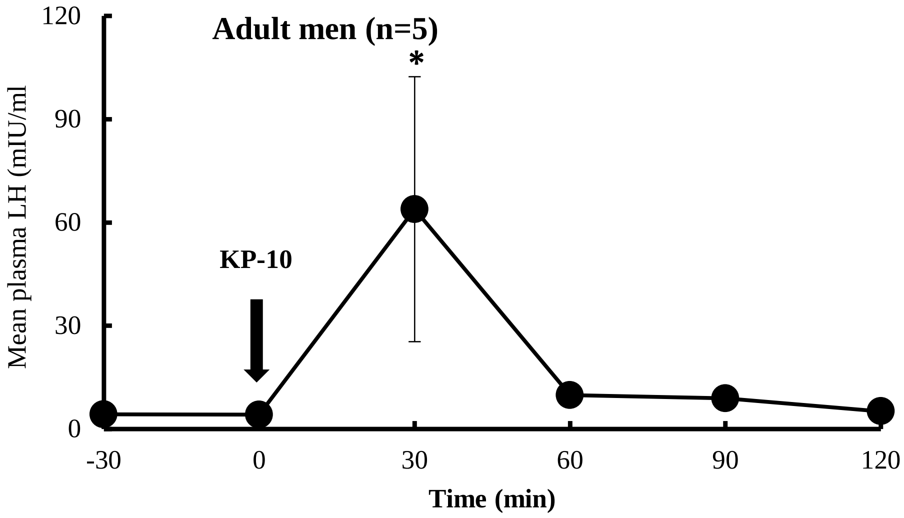

Figure 20: Mean±SEM plasma LH concentrations before and after administration of kisspeptin-10 (single iv bolus; ↓) in adult men (n=5). One-way ANOVA with repeated measures on log transformed data showed significant variation in LH levels at different times (*P<0.05 vs -30 min).

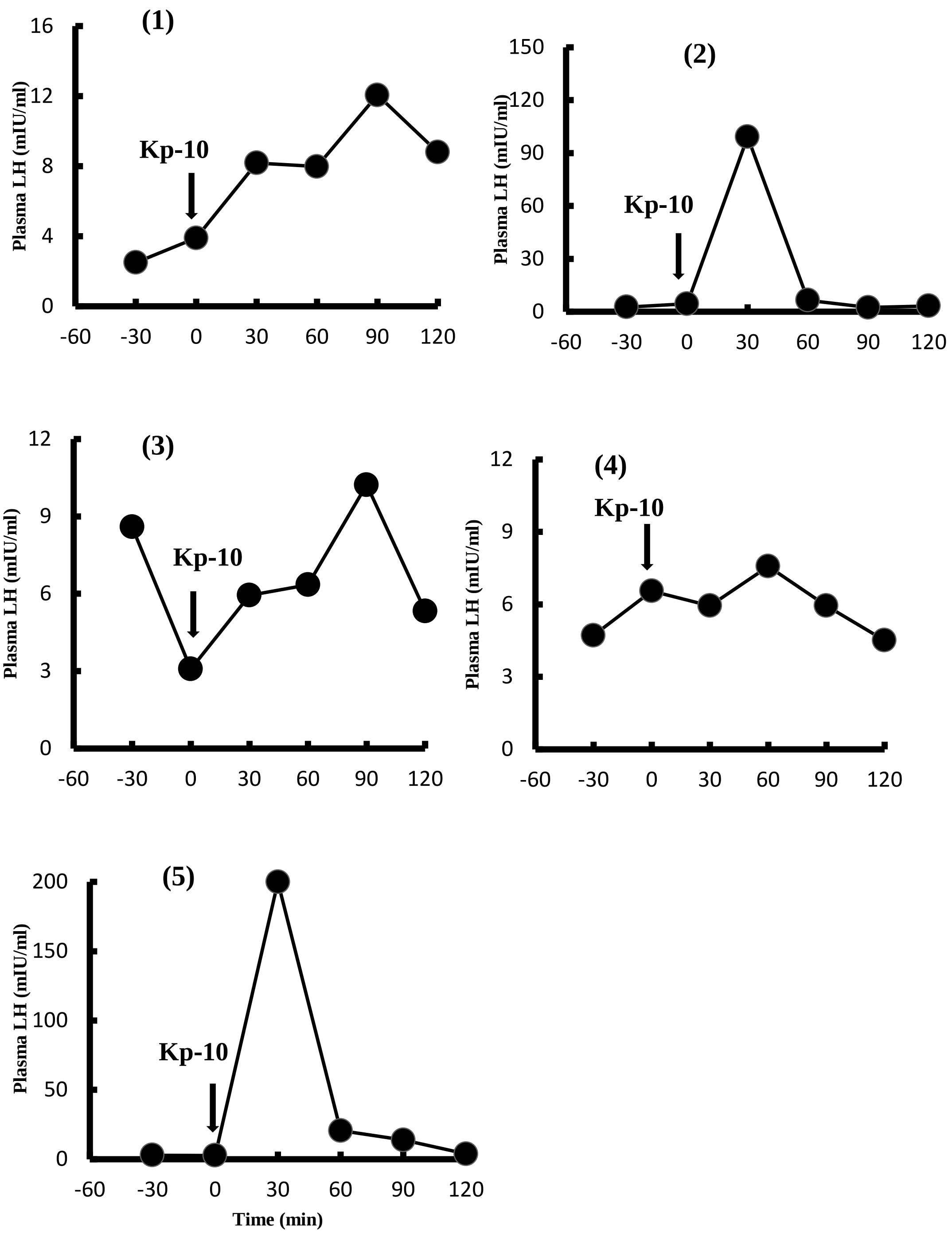

Figure 21: Plasma LH concentration before and after administration of kisspeptin-10 (single iv bolus; ↓) in individual adult men (1-5).

Plasma Testosterone levels in adult men at different time points

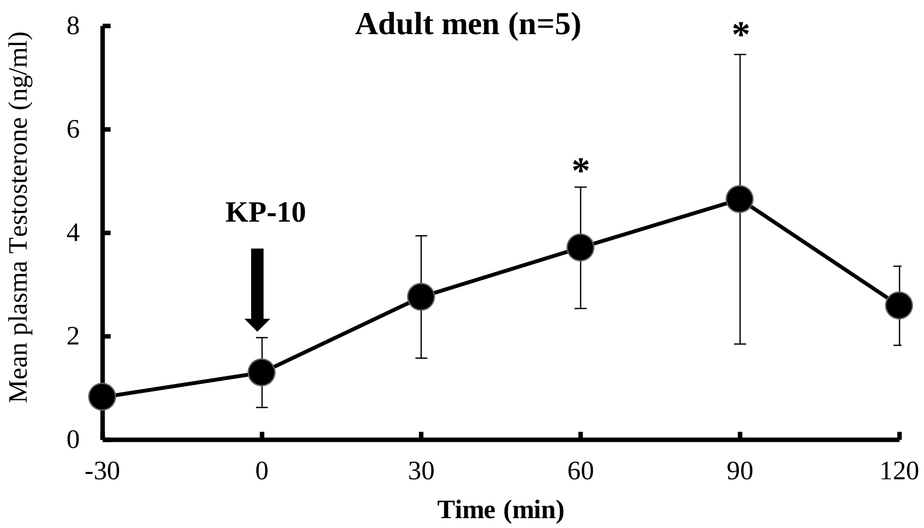

Figure 22: Mean±SEM plasma testosterone concentrations before and after administration of kisspeptin-10 (single iv bolus; ↓) in adult men (n=5). One-way ANOVA with repeated measures on log transformed data showed significant variation in testosterone levels at different times (*$P<0.05$ vs 0 min).

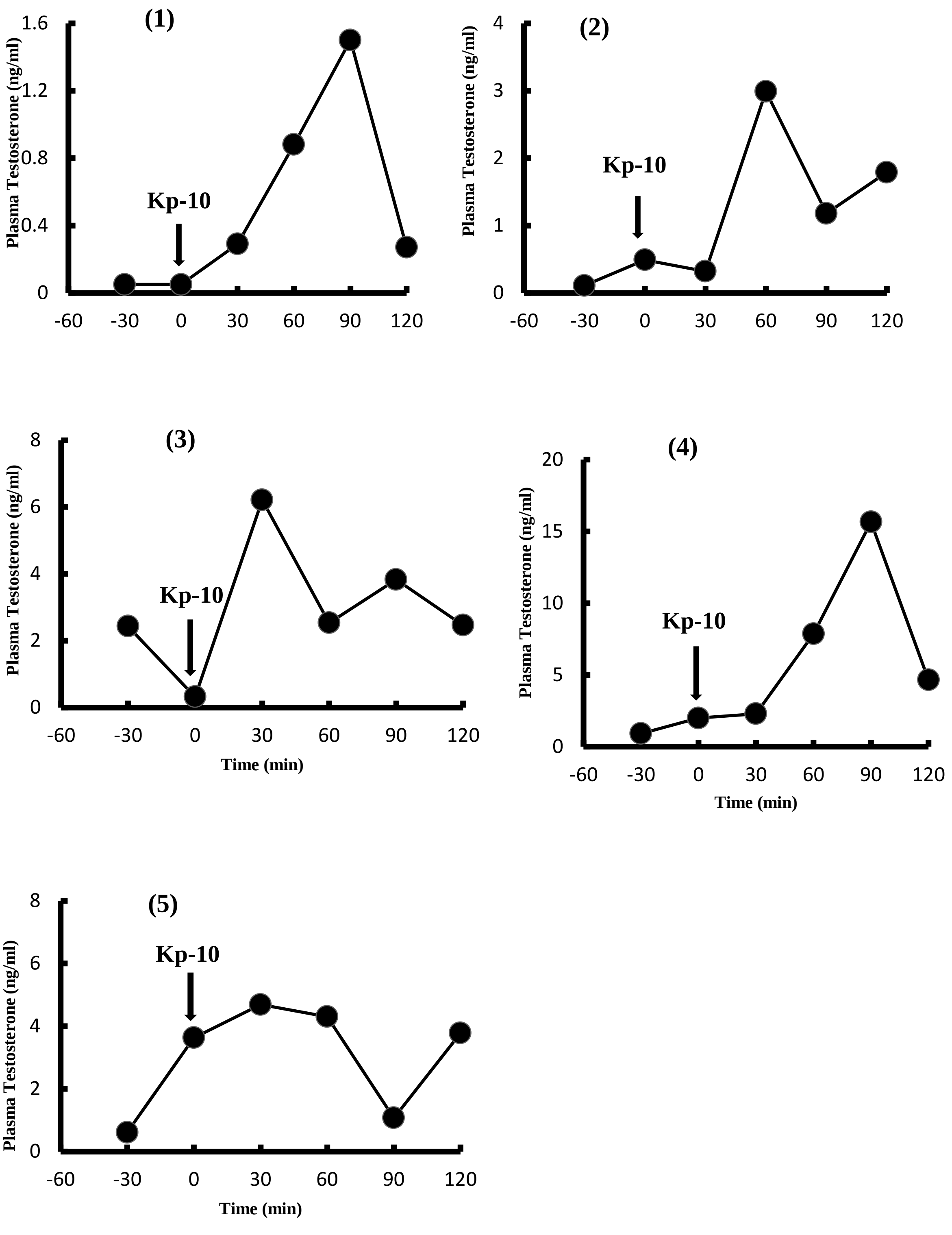

Figure 23: Plasma testosterone concentration before and after administration of kisspeptin-10 (single iv bolus; ↓) in individual adult men (1-5).
